# Efficient AntiMycolata Agents by Increasing the Lipophilicity of Known Antibiotics through Multicomponent Reactions

**DOI:** 10.3390/antibiotics12010083

**Published:** 2023-01-03

**Authors:** Angela Trejo, Carme Masdeu, Irene Serrano-Pérez, Marina Pedrola, Narcís Juanola, Ouldouz Ghashghaei, Guadalupe Jiménez-Galisteo, Rodolfo Lavilla, Francisco Palacios, Concepción Alonso, Miguel Viñas

**Affiliations:** 1 Departamento de Química Orgánica I, Facultad de Farmacia, Universidad del País Vasco/Euskal Herriko, Unibertsitatea (UPV/EHU), Paseo de la Universidad 7, 01006 Vitoria-Gasteiz, Spain; 2 Laboratory of Molecular Microbiology & Antimicrobials, Department of Pathology & Experimental Therapeutics, Medical School, University of Barcelona and IDIBELL, Feixa Llarga, s/n, 08907 Hospitalet de Llobregat, Spain; 3 Laboratory of Medicinal Chemistry, Faculty of Pharmacy and Food Sciences and Institute of Biomedicine (IBUB), University of Barcelona, Av. de Joan XXIII, 27-31, 08028 Barcelona, Spain

**Keywords:** antibiotics, drugs, multicomponent reactions, mycobacteria, resistant bacteria

## Abstract

New antibiotic agents were prepared using Povarov and Ugi multicomponent reactions upon the known drugs sulfadoxine and dapsone. The prepared derivatives, with increased lipophilicity, showed improved efficiency against Mycolata bacteria. Microbiological guidance for medicinal chemistry is a powerful tool to design new and effective antimicrobials. In this case, the readily synthesized compounds open new possibilities in the search for antimicrobials active on mycolic acid-containing bacteria.

## 1. Introduction

The performance and clinical use of many antibiotics, despite displaying high potency against the target, are severely diminished due to their poor absorption by the targeted microorganisms. This is particularly important in some bacteria, which feature an external lipophilic layer that further prevents the penetration of many drugs. Incidentally, serious infections are caused by bacteria belonging to this class; particularly the Mycolata group which is causing severe health problems: for instance, tuberculosis (*Mycobacterium tuberculosis*, MTB). In this respect, although some drugs are routinely used to treat the disease, there is an urgent need to improve their efficiency. We have focused on two targets: dihydropteroate synthase (DHPS) and dihydrofolate reductase (DHFR), which play important physiological roles in the survivability of bacteria, and we decided to modify known antibiotics to increase their penetration, basically keeping their original activity. 

Sulfonamides are the drugs of choice to monitor the growth and proliferation of MTBs by inhibiting the activity of DHPS and DHFR, which could explain the mechanism of action of these molecules. Mycolata is a broad and diverse group of mycolic acid-containing actinomycetes that includes a large number of species. Common to all these bacteria is the hydrophobic mycolic acid layer on the surface of the cells. The mycolic acids are covalently bound to the peptidoglycan of the bacterial cell wall [1]. The chain length varies considerably within the species containing those mycolic acids. Some particularly long mycolic acids are characteristic of mycobacteria (60 to 90 carbon atoms), while those that can be considered medium size are present in *Nocardiaceae* and related genera (46 to 58 carbon atoms), and small mycolic acids may be found in rhodococci (30 to 54 carbon atoms) and corynebacteria (22 to 38 carbon atoms) [2,3,4]. Other than mycolic acids, the cell wall of mycolata also may contain free lipids, such as trehalose dimycolates, glycosyl monomycolates, and peptidolipids [5]. Both free lipids and truly mycolic acids are arranged perpendicular to the cell surface, forming a membrane-like leaflet. It has been pointed out that the presence of mycolic acids on the surface of this vast group of bacteria highly restricts the entry of antibiotics and is subsequently one of the characteristics underlying intrinsic antimicrobial resistance [5]. Mycolata comprises many genera of clinical interest. Among them, the genus *Gordonia* belongs to the family *Gordoniaceae*, which is included in the suborder Corynebacteriales. *Gordonia* is gram-positive, as a very special characteristic, it is partially acid–alcohol fast [6]. The genus is catalase-positive, strictly aerobic; chemoorganotrophic cells are coccobacilli and contains a high G + C level (63–69%) [7,8]. *Gordonia* is closely related to *Nocardia*, although it does not produce mycelial forms, and some biochemical tests allow an easy differentiation between the two genera. *Gordonia* may be differentiated from *Rhodococcus*, another related bacterium, on the basis of 16S rRNA [9]. Some *Gordonia* species are relevant since they may act as opportunistic human pathogens. Isolations from clinical samples, often associated with implants, heart valves, or stents, are relatively frequent [10,11]. The bacterium may cause systemic infections in immunocompromised patients and local infections in immunocompetent patients. *G. jacobaea* was described in Spain in 2000 [12]. Thereafter, its genome was sequenced and annotated by us [13] and we investigated some of their proteins forming channels through the mycolic acid level [14]. According to the results, the closest related species is *G. sputi*. Recently, some reports have indicated its pathogenic ability [15].

The genus *Nocardia* contains aerobic actinomycetes, as well as catalase-positive, and gram-positive bacilli, and has branching filamentous forms [16]. *Nocardia* species can cause pulmonary infections, which are the most commonly reported for this bacterium. Moreover, they are responsible for cutaneous infections, which may also disseminate to other body regions. It has been pointed out that *Nocardia* is an opportunistic pathogen that mostly affects immunosuppressed patients [17]. Nevertheless, the treatment of nocardiosis is extremely complicated and requires further investigation [18]. *N. cyriageorgica* is a bacterium group harboring medium-length mycolic acids. Previously included in the *N. asteroides* complex, it was recently differentiated as an independent species due to molecular tests. The bacterium is commonly found as the etiological agent of nocardiosis [19]. This infection is becoming progressively important in serious and severe pulmonary infections and is particularly worrisome in immunocompromised individuals. The most relevant genus of mycolic acid containing bacteria is *Mycobacterium*. The best-known pathogen of this genus is *Mycobacterium tuberculosis*, the “Koch bacillus”. Nevertheless, other species known as non-tuberculous mycobacteria (NTM) are increasingly recognized as relevant human pathogens causing infections even in animals [20,21]. Generally, NTM are classified into two groups, the so-called slow growers and rapid growers, depending on their rate of growth. *M. abscessus abscessus*, a rapid-growing mycobacteria belonging to the *M. abscessus* complex, has been shown to be the most frequently encountered causative agent in human infections, accounting for approximately 65–80% of rapid-growing mycobacterial respiratory diseases. This bacterium is highly resistant to many antibiotics, which is why treatments are often not capable of eliminating the infectious agent. It is mainly found in water-related environmental sources such as swimming pools and water tanks, although it can be found in other places such as livestock, seawater, and medical devices [20]. Therefore, exposure to this bacterium is very common, making it the main etiological agent of pulmonary infections caused by rapidly growing NTM. Intrinsic and acquired resistance to conventional anti-mycobacterial agents have been associated with this strain [21].

**Figure 1:**
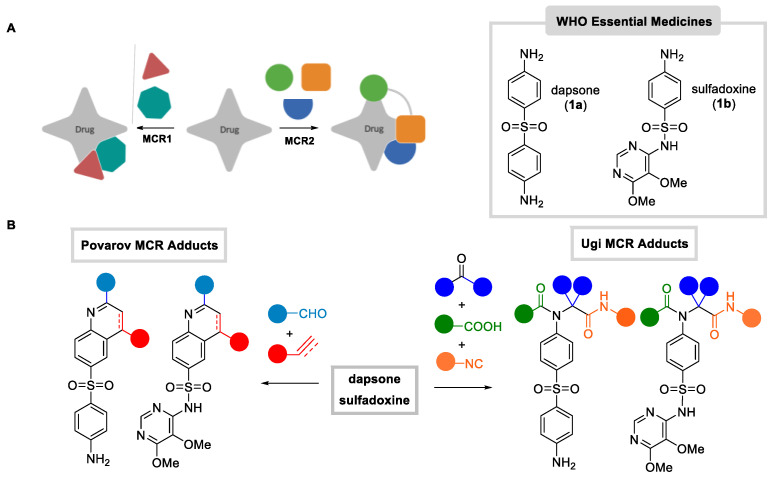
(**A**) Drugs from Drugs through MCRs. (**B**) Ugi and Povarov reactions with dapsone and sulfadoxine to yield active derivatives.

The modification of existing drugs is a powerful way to develop new pharmacologically active derivatives and has been successfully taken up by the pharma industry. In this respect, the Late-Stage Functionalization approach is appealing for the direct, situ-selective introduction/modification of certain connectivities found in drugs [22]. This methodology may be particularly relevant in the development of new antibiotics, an especially tough endeavor [23]. Recent examples include a programmed and selective structural modification of complex compounds to improve their potency [24,25], and even to repurpose different drugs for antibiotic activity [26]. In a previous work, we promoted a more drastic transformation, based on the reactivity of functional groups present in the structure of the drug through Multicomponent Reactions (MCRs, Figure 1A). In these processes, three or more substrates interact to form a single adduct in one-step, forming alongside several bonds [27]. In this way, we modified trimethoprim (TMP) through the Groebke-Blackburn-Bienaymé MCR [28] to rapidly yield a small library of TMP derivatives, with some of them displaying a similar potency but with a faster mode of action [29]. Furthermore, it was later shown that the docking of these compounds in the target, drastically modified the binding mode of the original drug, invading the site occupied by the cofactor, opening in this way new avenues for structure-guided modifications [30].

In this context, we decided to start a programmed search of the chemical space around well-established drugs rather than trying to find new hits by screening large, diversified libraries [31]. Trying to expand this type of exploration around potent hits, we chose a couple of WHO-listed essential medicines, namely the antibiotics dapsone (**1a**) and sulfadoxine (**1b**), which display in their structure aniline residues capable of undergoing general and useful MCRs (Figure 1A). The viability of the approach relies on the following requirements: i) the initial drug should have functional groups amenable to participate in MCRs; and ii) the obtained adducts should display meaningful interactions with the target to justify binding, although in an altered manner. This last point is key for the improvement of the activity profile (potency, resistance, PK, etc.). Incidentally, it is important to mention that in the present case, we intentionally sought to increase lipophilicity to facilitate crossing the membranes in mycolata. Intuitively, both the Ugi and Povarov MCRs accomplish these points in a simple and straightforward way. Thus, we planned and executed these MCRs, widely used in medicinal chemistry [32], involving the aniline moiety present in both drugs. In particular, we promoted Ugi MCRs with carbonyls (aldehydes and ketones), isocyanides and carboxylic acid partners [33], Povarov MCRs with aldehydes and electron rich olefins [34,35,36], or acetylenes (Figure 1B) [37].

Then, we explored the biological activity of these MCR derivatives on the three mentioned representatives of mycolata, one belonging to the short mycolic acid group (*G. jacobaea*), one of the groups of medium length (*N. cyriacigeorgica*), and one harboring long mycolic acids (*M. abscessus*), aiming to explore their antimicrobial capabilities.

## 2. Results & Discussion

### 2.1. Chemical Synthesis

**Figure 2:**
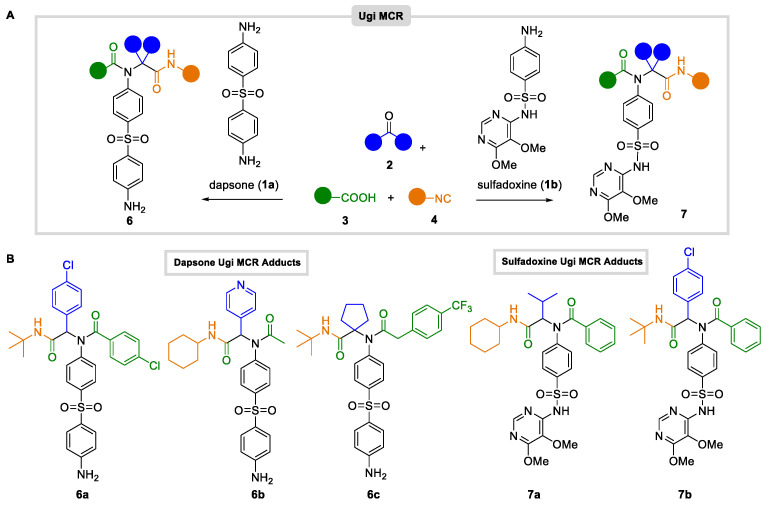
(**A**) Ugi MCR transformations with dapsone and sulfadoxine. (**B**) Collection of Ugi-derived dapsone (**6a**–**c**) and sulfadoxine (**7a**,**b**) MCR adducts.

First, we analyzed the Ugi MCRs transformation of dapsone **1a** and sulfadoxine **1b** with carbonylic compounds (aldehydes and ketones **2**), carboxylic acids **3**, and isocyanides **4**, under typical conditions (MeOH solution). In this manner, a collection of derivatives **6** and **7** was obtained (Figure 2A). All compounds were suitably prepared (unoptimized procedures) and purified, usually through automated chromatography (Figure 2B). In this way, both drugs (**1a** and **1b**) yielded the corresponding Ugi adducts as enantiomeric mixtures (**6a**, **6b**, **7a**, and **7b**) or single compounds (**6c**), which were tested once purified.

**Figure 3:**
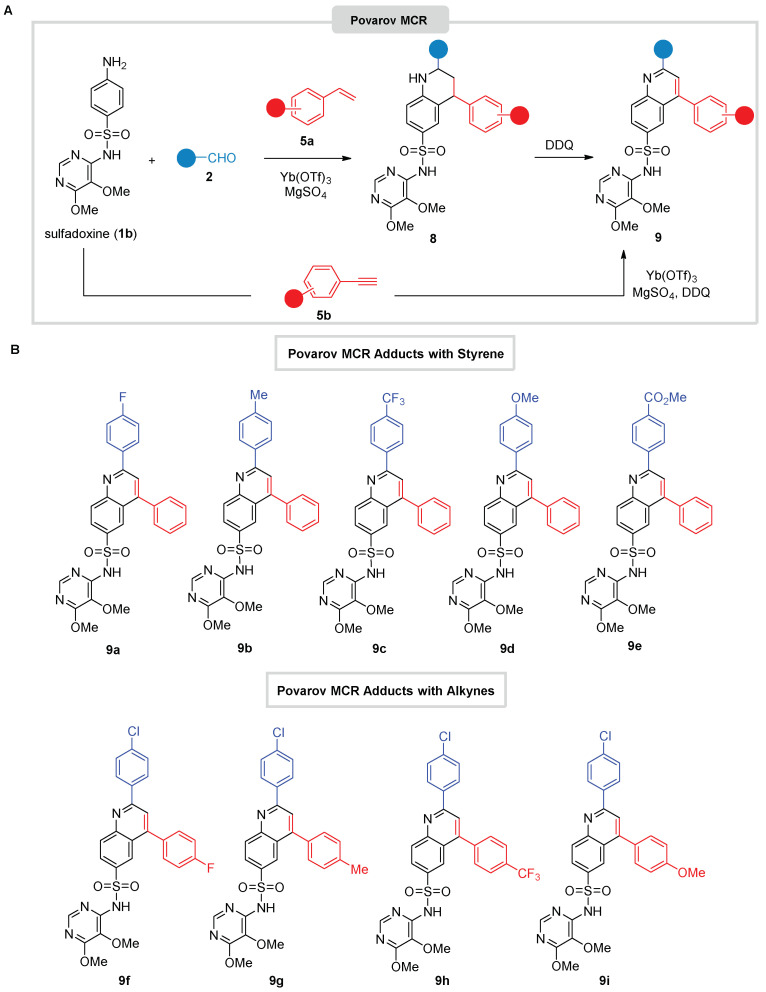
(**A**) Povarov MCR transformations with sulfadoxine. (**B**) Collection of Povarov-derived sulfadoxine MCR adducts **9**.

Next, we tackled the Povarov modification of the substrates with styrenes. Dapsone (**1a**) was troublesome, likely due to its limited solubility in the standard reaction conditions (ACN, Yb(OTf)_3_ catalysis, MgSO_4_) [38]. Pleasantly, sulfadoxine **1b** was much more productive and produced a wide variety of Povarov adducts, involving several aldehydes (benzaldehyde and a range of substituted derivatives) and activated alkenes (styrenes) (Figure 3A). At this point, tetrahydroquinoline derivatives **8** were obtained as major products (usually with a *cis* stereochemistry), although small amounts of the aromatized derivatives **9** were also observed. Subsequent purification (SiO_2_ column chromatography) always produced an inseparable mixture of tetrahydroquinoline **8** and quinoline derivatives **9**. Therefore, after consuming the starting materials in the Povarov MCR, sulfadoxine **1a**, aldehyde **2** and styrenes **5a**, we proceeded to the in-situ aromatization of tetrahydroquinoline derivatives **8**, without formal isolation and characterization at this level, by treatment with DDQ following published protocols (Figure 3A) [36]. In this way, a library of complex fused quinolino-sulfadoxine derivatives **9** was readily prepared (Figure 3B). Finally, to synthesize derivatives **9** in a shorter and more efficient way, a variation of the Povarov reaction using phenylacetylenes **5b** as a dienophile source in the presence of DDQ directly afforded the oxidized adducts **9** [39] in somewhat low yields through unoptimized procedures (see Supplementary Materials).

The aforementioned compounds were forwarded for antimicrobial studies. Moreover, their structures were submitted to the SwissADME website [40] to preliminarily estimate some physicochemical descriptors and ADME parameters. The calculated properties showed good compliance with Lipinski rules (usually 0, 1 violations), increased lipophilicity, low GI absortion, and no BBB permeability (see Supplementary Materials).

### 2.2. Microbiological Studies

**Figure 4:**
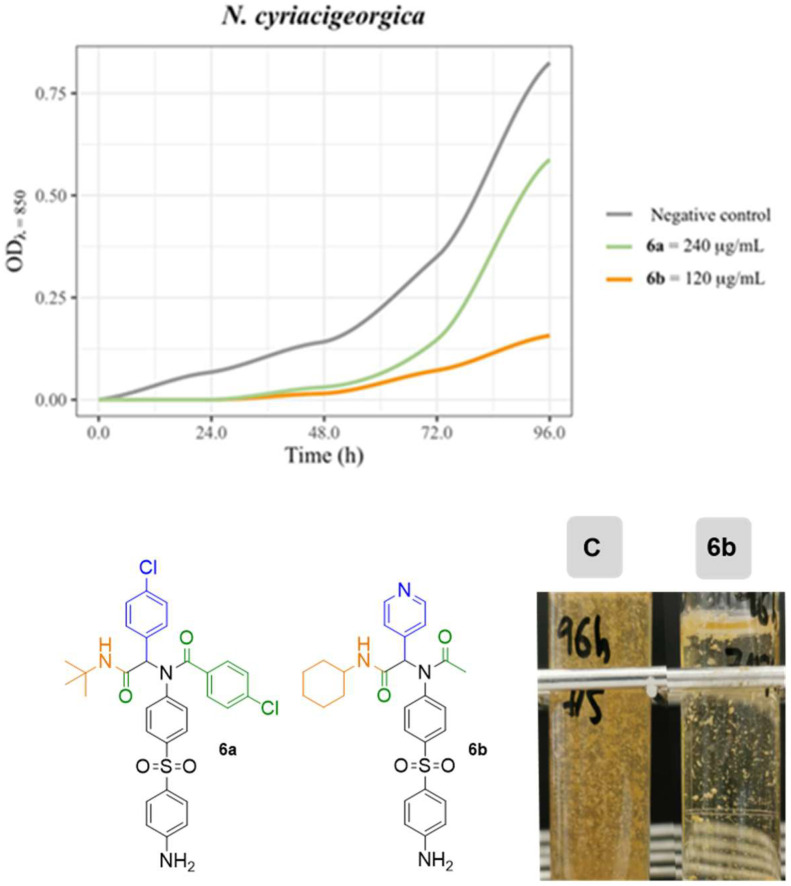
Comparative cultures of *N. cyriacigeorgica* negative control (C) and in the presence of **6b**. In the growth curves, **6b** completely inhibited growth in the first 48 h. Negative control corresponds to a culture lacking antibiotic and with the same concentration of DMSO. Graphic drawn in R with the ggformula and ggplot2 packages [41,42].

Initial experiments were conducted to explore the eventual antimycolata capacity of the synthesized molecules. After 48 h of incubation, although **1a** and **1b** do not form inihibition zones, several molecules displayed activity on the plates inoculated with N. cyriacigeorgica. Only compound **6a** was moderately active on *G. jacobaea* MV-1, whereas none of them decreased the growth of *M. abscessus* (Table 1). Moreover, the dapsone derivative **6b** failed to inhibit the growth of *N. cyriacigeorgica* at the concentration used for the rest of the compounds, although it was able to inhibit the growth at 120 µg/mL in an effective manner during long periods of incubation (Figure 4).

**Figure 5:**
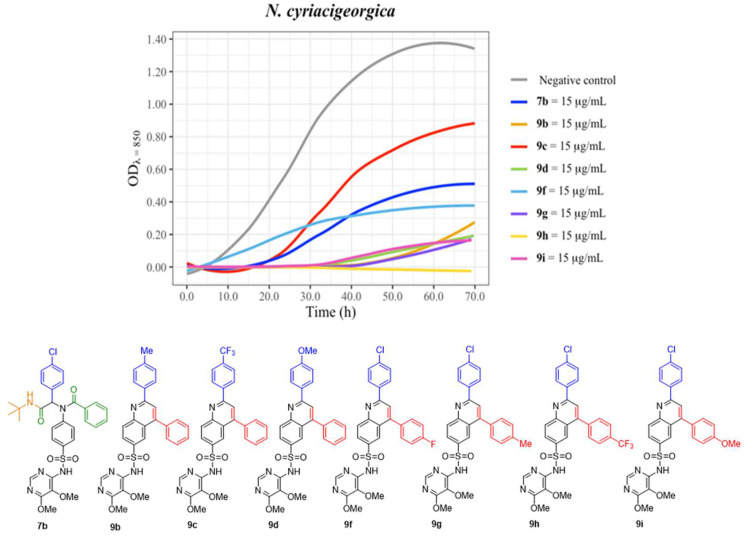
*N. cyriacigeorgica* growth curves. Fresh cultures were inoculated in CAMHB, and several conditions were tested: the negative control (grey) and the compounds at 15 μg/mL. The bacterial growth was measured by optical density (OD) for 70 h. A comparative graph of all tested products. Negative controls correspond to a culture lacking antibiotic and with the same concentration of DMSO. Graphics drawn in R with the ggformula and ggplot2 packages [41,42].

As an initial assessment, the two most active sulfadoxine derivatives on the disk-diffusion test on *N. cyriacigeorgica* were selected (Table 1) to draw growth curves and thus, to obtain data of their activity over time. These compounds (**9c** and **9f**) were tested first in *N. cyriacigeorgica* cultures at a 15 μg/mL concentration [≈25 μM]. This concentration was preliminary chosen being lower than the one used for disk-diffusion tests (25 μg/disk). In these growth curves (Figure 5), **9f** greatly reduced the overall growth of *N. cyriacigeorgica*, while surprisingly, **9c** did not affect the bacterial growth. (Figure 5). On the other hand, the dapsone-derived **6a** product was also studied, showing moderate activity. In these experiments, **1a** and **1b** at the same concentrations that synthesized compounds do not alter the growth curves.

Derivatives **7b**, **9b**, and **9d** significantly reduced bacterial growth. Compounds **9b** and **9d** featured the greatest reduction in bacterial growth and displayed a 20-hour longer lag phase compared with the negative control. Compound **7b** also reduced the bacterial growth, although to a lesser extent, and did not increase the lag phase.

Lastly, compounds **9g**, **9h**, and **9i** were tested under the same conditions (Figure 5) and all showed an effect on the bacterial growth. Derivative **9h** completely inhibited the bacterial growth in the first 70 h. This behavior suggests that **9h** may act as a bactericidal agent since if some surviving individuals are there sooner or later, one would expect growth. This is going to be investigated. Finally, compounds **9g** and **9i** reduced growth significantly to the same extent, both greatly prolonging the lag phase (20 h).

These growth kinetics suggested that compounds **7b**, **9b**, **9d**, **9f**, **9g**, and **9i** probably act as effective inhibitors of bacterial growth for long periods of time, although regrowth was seen, demonstrating that the complete elimination of bacteria was not accomplished. In fact, regrowth at long periods of incubation is found in almost all antimicrobials, irrespective of their targets and kinetics of action, having an inconclusive meaning, since it is strongly influenced by factors such as the alterations of the molecule or of its concentration itself, heteroresistance, mutational phenomena, etc.

**Figure 6:**
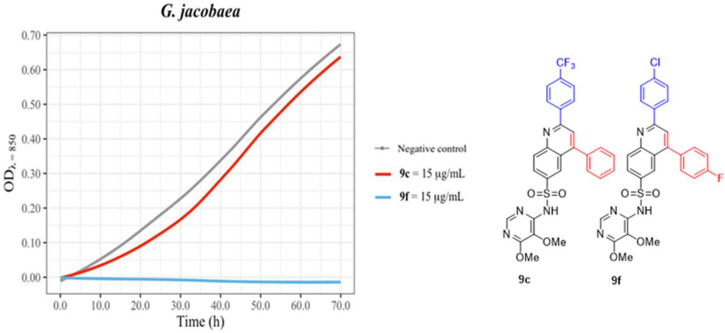
*G. jacobaea* growth curves. Fresh cultures were inoculated in CAMHB, and several conditions were tested: the negative control (grey), and the **9c** (red) and **9f** (Blue) at 15 μg/mL. The bacterial growth was measured by optical density (OD) for 70 h. The other compound related to **9c** and **9f** is **9h** whose results were indistinguishable from those of **9f**. Negative control corresponds to a culture lacking antibiotic and with the same concentration of DMSO. Graphics drawn in R with the ggformula and ggplot2 packages [41,42].

The assay of selected derivatives on *G. jacobaea* demonstrated that, in spite of the lack of visible effect on disk diffusion on plates, the behavior in a liquid environment was completely different, at least for some of them. Figure 6 shows the growth kinetics of *G. jacobaea* in the presence of **9f** and a complete lack of effect for compound **9c**.

These results suggest that some of the derivatives tested can potentially be considered promising agents for the treatment of infections caused by short mycolic acids containing mycolata. Moreover, they also call into question the estimation of sensitivity to antimicrobials simply by disk diffusion in solid media plates or even MIC determination for bacteria with strong hydrophobic components on their surfaces or displaying very low rates of growth. If conclusions had only been drawn from the activity of these products as determined by disk-diffusion, the real antimicrobial activity of **9f**, which fully suppressed growth for at least 70 h, would have been ignored.

**Figure 7:**
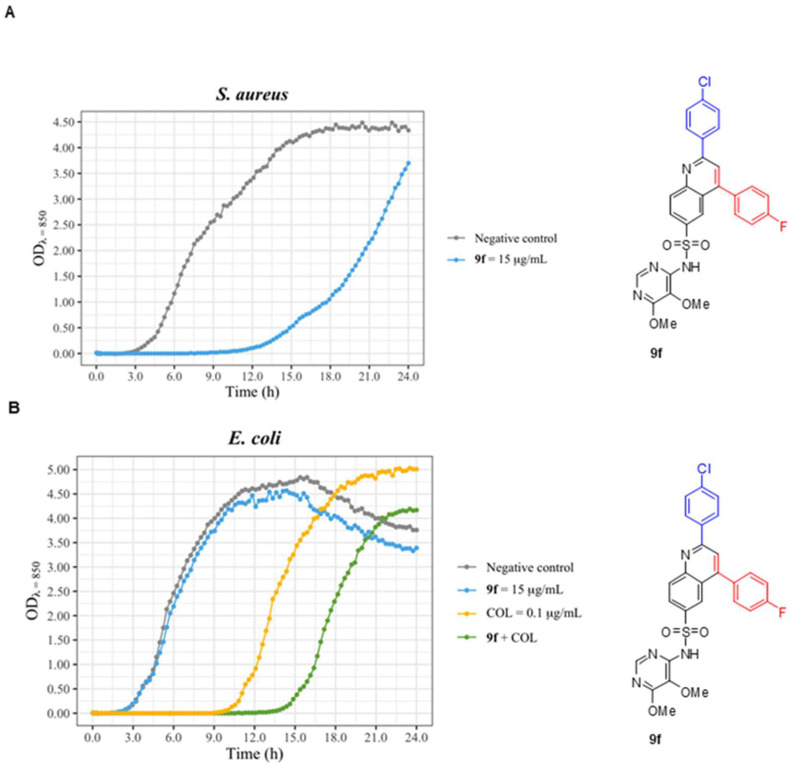
(**A**) *Staphylococcus aureus* growth curve. A fresh culture of *S. aureus* ATCC 29213 was inoculated in CAMHB, and two conditions were tested: the positive control (grey) and **9f** at 15 μg/mL (light blue). (**B**) Fresh cultures of *E. coli* ATCC 29552 were inoculated in CAMHB, and several conditions were tested: negative control (grey) and **9f** at 15 μg/mL, (light blue) colistin 0.1 μg/mL (yellow), and a combination of **9f** at 15 μg/mL and colistin 0.1 μg/mL (green). The bacterial growth was measured by optical density (OD) for 24 h. Negative control corresponds to a culture lacking antibiotic and with the same concentration of DMSO. The graphic drawn in R with the ggformula and ggplot2 packages [41,42].

After analyzing *N. cyriacigeorgica*’s growth curves with the different studied compounds, **9f** was selected for further experiments, as this compound was considered to have the most potential interest. Initially, the eventual synergy with TMP was explored. No synergy was found between the compounds **9f** and TMP. Another relevant aspect of **9f** characteristics is its spectrum of activity. Thus, growth curves were drawn for *S. aureus* ATCC 29213 and *E. coli* ATCC 29552strains as representatives of conventional Gram-positive and Gram-negative bacteria respectively, both extensively used in antimicrobial research (Figure 7). In *S. aureus* ATCC 29213 cultures, derivative **9f** at 15 μg/mL acted by prolonging the lag phase for approximately 10 h, and thus, in 24 h, the culture did not reach the stationary phase (Figure 7A).

**Figure 8:**
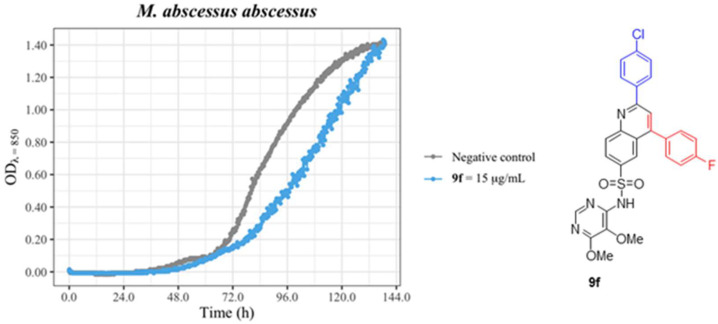
*M. abscessus* abscessus growth curves. Fresh curtures were inoculated in CAMHB and two conditions were tested: the negative control (grey) and **9f** (blue) at 15 μg//mL. The bacterial growth was measured by optical density (OD) for 144 h. Negative control corresponds to a culture lacking antibiotic and with the same concentration of DMSO. The graphic drawn in R with the ggformula and ggplot2 packages [41,42].

The experiments conducted in *M. abscessus* demonstrated that, in the actual state, this family of compounds failed in interfering with the capability of growth, as can be concluded from Figure 8. A new chemical modification of these structures is in course to attempt the synthesis of active molecules on long mycolic acids containing bacteria.

In order to investigate potential modifications in the spectrum of action, experiments were conducted in standard conventional bacteria, such as *Staphylococcus aureus* ATCC 29213 and *Escherichia coli* ATCC 29552. The active products were able to inhibit the growth of gram-positive bacterium as can be concluded from Figure 7.

Noteworthy, in *E. coli* ATCC 29552 cultures, compound **9f** failed in affecting bacterial growth (Figure 7B). To test whether this lack of effect was due to the presence of the typical outer membrane of gram-negative bacteria, additional experiments were conducted to draw growth curves of *E. coli* ATCC 29552 with a combination of colistin and derivative **9f**. The hypothesis to be tested was if compound **9f** could enter the bacteria thanks to the severe alteration that colistin produces on the bacterial outer membrane. Figure 7B shows the effect on the bacterial growth of **9f**, colistin, and their combination. As previously mentioned, **9f** alone does not alter the growth, colistin alone prolongs the lag phase for 9 h, and the combination of both prolongs the lag phase up to almost 15 h (6 hours more than colistin alone). In spite of this moderate prolongation of the lag phase, the results seem to demonstrate that the product was not (or poorly) active on gram-negatives, and that probably this poor activity is not due to the impermeability of outer membrane. In any case, the increase in lipophilicity of the antibiotic molecules can open a new route of entry (the lipid route) independent from porins. This may explain the synergism existing between **9f** and colistin that prolong the bacteriostatic effect for 6 h even though compound **9f** alone has no effect. A preliminary inspection on the structural features of the tested compounds suggests that the increased lipophilicity of the synthesized derivatives positively contributes to their activity upon mycolata (see LogP data in Supplementary Materials SWISSADME profile for all new compounds). For instance, note the increased consensus Log P values for compound **9h** (5.68) vs. **9f** (4.97) and for derivative **9c** (5.13) vs. analogue **9a** (4.49), which basically seems to support the tendency. Notably, the substitution pattern upon both aromatic rings of the Povarov adducts **9** seems to determine the potency levels, likely arising from favored binding modes in the active site of the targets. In this way, in the prepared minilibrary, clear differences appear among distinct residues in the Povarov sulfadoxine series. For instance, changing a CH_3_ substituent (**9g**) for a CF_3_ residue (**9h**) on the upper phenyl ring (aldehyde moiety) results in a remarkable inhibition of bacterial growth (Figure 5). Additionally, a deep gain can be observed when going from an F atom on compound **9a** to a CF_3_ substituent on **9c**, affecting in this case to the lower phenyl ring (dienophile moiety) (Table 1). After these initial results, systematic S.A.R. studies may be conducted upon selected derivatives to further optimize their activities. Moreover, a computational approach upon the isolated targets may predict the main structural modifications needed to tune the newly described scaffolds.

In conclusion, we have developed a direct chemical approach for the synthesis of lipophilic derivatives of dapsone and sulfadoxine though two types of MCRs (Ugi and Povarov transformations) and tested them against a variety of mycolata bacteria. Some derivatives, especially **9f** and **9c**, showed an increased activity upon these microorganisms and the results open many possibilities to be further explored along these lines. Moreover, it should be taken into account that experimental work with slow-growing bacteria is a notable handicap when considering the use of antimicrobials, since long incubation periods obviously create conditions in which the antibiotic can most likely be altered. It is therefore essential to perform experiments parallel to those referred to here, what would happen if we were to ensure a constant antimicrobial concentration over time by adding at determined times (for example, every 8 h) an amount of antibiotic that would maintain the levels stable. These experiments are currently underway.

## 3. Materials and Methods

### 3.1. Chemical Synthesis

The preparation of Ugi derivatives was performed using dapsone **1a** or sulfadoxine **1b**. A mixture of dapsone **1a** (1.0 mmol, 1.0 eq.) or sulfadoxine **1b** (1.0 mmol, 1 eq.), carbonylic compound **2** (1.0 mmol, 1.0 eq.), and carboxylic acid **3** (1.0 mmol, 1.0 eq.) were added to a Schlenk flask with 2.0 mL of MeOH. The reaction mixture stirred at room temperature for 10 min. Then the suitable isocyanide **4** (1.0 mmol, 1.0 eq.) was added into the mixture and the reaction was left stirring overnight at room temperature. After reaction completion was determined by LC-MS or TLC, the reaction mixture was evaporated under reduced pressure. The reaction crude was diluted with EtOAc (20 mL) and was mixed with saturated aqueous solution of NaHCO_3_. The aqueous phase was separated and extracted with EtOAc (2 × 20 mL). The combined organic phases were dried with MgSO_4_, filtered, and evaporated under reduced pressure. The pure products **6** and **7** were obtained through automated flash chromatography.

For Povarov transformations with styrenes, a solution of sulfadoxine **1b** (1.0 mmol, 1.0 eq.), aldehyde **2** (1.0 mmol, 1.0 eq.), and ytterbium triflate (20% mmol) in 5 mL of dry acetonitrile, and in presence of anhydrous MgSO_4_ (800 mg), the corresponding dienophile **5** (1.5 mmol, 1.5 eq.) was added. The resulting mixture was stirred under a nitrogen atmosphere at room temperature until TLC analysis indicated the disappearance of the starting materials. The solution was then diluted with dichloromethane (20 mL), washed with water (2 × 10 mL), and the aqueous layer was again extracted with dichloromethane (2 × 10 mL). The organic phase was dried over anhydrous MgSO_4_, filtered, and concentrated under a vacuum. The resultant crude residue was dissolved in CHCl_3_ (15 mL), and DDQ (2 mmol) was added to the solution. The reaction mixture was stirred overnight at room temperature in an open vessel. A saturated aqueous solution of NaHCO_3_ (10 mL) was added, the resulting mixture was extracted with dichloromethane (2 × 25 mL), dried over anhydrous MgSO_4_, filtered, and concentrated under reduced pressure. The crude residue obtained was purified by flash column chromatography on silica gel (hexane-ethyl acetate 80:20) to afford the dehydrogenated compounds **9**.

For Povarov transformations with acetylenes, sulfadoxine **1b** (0.483 mmol, 0.150 g, 1 eq.), aldehyde **2** (0.483 mmol, 1 eq.), and ytterbium triflate (0.097 mmol, 0.06 g, 20 % mmol) were dissolved in 5 mL of dry acetonitrile, in the presence of anhydrous MgSO_4_ (400 mg). The corresponding phenyl acetylene **5** (0.7245 mmol, 1.5 eq.) and DDQ (0.483 mmol, 0.109 g, 1 eq.) were added to the suspension at room temperature. The mixture was then stirred under nitrogen at the suitable temperature until TLC analysis indicated the consumption of the starting materials. The contents were added to a saturated aqueous solution of NaHCO_3_ (10 mL) and extracted with dichloromethane (2 × 10 mL). The organic phase was dried over anhydrous MgSO_4_, filtered, and concentrated under a vacuum. Removing the solvent, the crude obtained was purified by column chromatography on silica gel using an elution gradient of hexane-ethyl acetate (80-20) to yield the pure products **9**.

Full details and characterization data of all new compounds can be found in the Supplementary Materials.

### 3.2. Culture Conditions

A total of 3 bacterial strains belonging to 3 different species of Mycolata were cultured for the study of the antimicrobial capacity of new synthetic molecules so called **7** and **9** series: *Gordonia jacobaea* MV-1 [12,13,14], *Nocardia cyriacigeorgica* (a clinical isolate; laboratory identification number: 13485883), and *Mycobacterium abscessus abscessus* (a clinical isolate gently provided by the Hospital de Bellvitge, Barcelona). To support certain experiments, bacterial strains *Escherichia coli* (ATCC 29552) and *Staphylococcus aureus* (ATCC 29213) were also cultured.

Bacteria were cultivated in TSB (Tryptic Soy Broth) medium in liquid and in TSA (Tryptic Soy Agar) medium in solid. Both *G. jacobaea* MV-1 and *N. cyriacigeorgica* were incubated at 30 °C, while *M. abscessus abscessus*, *E. coli* ATCC 29552, and *S. aureus* ATCC 29213 were incubated at 37 °C. In addition, the handling of *M. abscessus abscessus* cultures required a vertical laminar flow chamber (biological security) as a safety measure.

When performing susceptibility experiments, the media used were CAMHB (Cation-Adjusted Mueller-Hinton Broth) and CAMHA (Cation-Adjusted Mueller-Hinton Agar) for liquid and solid cultures, respectively. The CAMHA medium was prepared by supplementing Agar to CAMHB in its preparation.

### 3.3. Antimicrobial Compounds

All compounds were stored pure in the solid state at −20 °C. Thus, their solubilization was required before the susceptibility experiments. First, they were dissolved in pure DMSO at a concentration of 250 mg/mL to prepare a first stock from which serial dilutions were made in distilled water 1:10 (compound: H_2_O; *v/v*) for susceptibility experiments.

### 3.4. Susceptibility Measurements

(A) disk diffusion: to determine the eventual antimycolata activity of molecules, disc-diffusion determinations of the susceptibility of *G. jacobaea* MV-1 and *N. cyriacigeorgica* were performed. CAMHA plates were inoculated with each bacterial species starting from two-day liquid cultures in TSB. Next, 10 μL of a dilution of the molecule at 2.5 mg/mL was loaded onto Watman paper disks such that 25 μg of the molecule was present on each disk. These disks were placed on the plates and incubated at 30 °C for approximately 48 h. (B) Growth curves: Once the antimicrobial capacity of molecules was detected on plates, growth curves were drawn in the presence of the molecule by culturing and optical density control in individual bioreactors (RTS-1, Biosan, Latvia). Bioreactors require a minimum culture volume of 10 mL to allow measurements of bacterial growth by optical density (OD).

Starting from 24 h cultures of *N. cyriacigeorgica* and 24 h cultures of *G. jacobaea* MV-1 incubated in an orbital shaker at 200 rpm, aliquots of 100 μL were added to CAMHB, with the molecule at a final concentration of 15 μg/mL. For the rest of the bacteria assayed, starting bacterial cultures were as follows: 72 h cultures for *M. abscessus abscessus*, and O/N cultures for *S. aureus* ATCC 29213 and *E. coli* ATCC 29552 adjusted to 0.5 McFarland (OD_625_ = 0.08–0.12). These cultures were prepared in tubes, called TubeSpin^®^ Bioreactor 50–20 (Biosan, Latvia), whose stopper presents holes with a bacterial filter (0.2 μm pore) to allow air to reach the culture. Incubation was conducted at 30 °C under 200 rpm/sec orbital agitation to avoid bacterial sedimentation. Bacterial growth was quantified by an automatic register of OD at 850 nm/minute for the first 25 min, and then at 15-minute intervals thereafter. These first 25 min of measurement allow us to check if the cultures are in stable conditions. The cultures were incubated and measured for at least 70 h. Experiments were made at least in triplicate. Curves were obtained at 30 °C and 70 h *G. jacobaea and N. cyriageorgica;* 144 h at 37 °C for *M. abscessus abscessus*, and 24 h at 37 °C for *S. aureus* ATCC 29213 and *E. coli* ATCC 29552.

Analysis was conducted in R, and figures were produced using the packages ggformula [41] and ggplot2 [42]. Since the aerobic growth of *G. jacobaea* MV-1 and *N. cyriacigeorgica* in agitation promotes aggregates of varying sizes, thus disturbing the measurements, a non-parametric method (loess or lowess; locally weighted scatterplot smoothing) using the ggformula package [41] was applied to calculate and plot the curves.

## Figures and Tables

**Table 1:** Diameters of inhibition zones in CAMHA plates. Inhibition zones were measured after incubation at 30 °C for approximately 48 h.

Molecule	*G. jacobaea*	*N. cyriacigeorgica*	*M. abscessus*
**1a**	-	-	-
**1b**	-	-	-
**6a**	9.0 mm	11.0 mm	-
**6b**	-	-	-
**6c**	-	-	-
**7a**	-	10.0 mm	-
**7b**	-	10.0 mm	-
**9a**	-	-	-
**9b**	-	13.0 mm	-
**9c**	-	**15.0 mm**	-
**9d**		11.0 mm	-
**9e**	-	-	-
**9f**	-	**18.0 mm**	-
**9g**	-	11.5 mm	-
**9h**	-	8.5 mm	-
**9i**	-	14.0 mm	-

## Data Availability

Not applicable.

## Supplementary Materials

The following Supplementary Materials can be downloaded at: https://www.mdpi.com/article/10.3390/antibiotics12010083/s1. File S1: Full experimental details on the synthesis and characterization of all new products prepared. SWISSADME profile for these compounds in a separate file.
