# Supplementary material for: Efficient AntiMycolata Agents by Increasing the Lipophilicity of Known Antibiotics through Multicomponent Reactions
Source: Antibiotics (Basel). 2023 Jan 3;12(1):83. doi: 10.3390/antibiotics12010083 (PMC9854905; doi:10.3390/antibiotics12010083)

SUPPORTING INFORMATION

Efficient AntiMycolata Agents by Increasing the Lipophilicity of Known Antibiotics through Multicomponent Reactions

Angela Trejo[$1], Carme Masdeu[$1], Irene Serrano[$2], Marina Pedrola[3], Narcís Juanola[3], Ouldouz Ghashghaei[3], Guadalupe Jiménez-Galisteo[2], Rodolfo Lavilla[3]*, Francisco Palacios,[1] Concepción Alonso[1]*, Miguel Viñas[2]*

[1] Departamento de Química Orgánica I, Facultad de Farmacia, Universidad del País Vasco/Euskal Herriko, Unibertsitatea (UPV/EHU), Paseo de la Universidad 7, 01006 Vitoria-Gasteiz, Spain

[2] Laboratory of Molecular Microbiology & Antimicrobials, Dept. Pathology & Experimental Therapeutics, Medical School, University of Barcelona and IDIBELL, Feixa Llarga, s/n. 08907 Hospitalet de Llobregat (Barcelona), Spain

[3] Laboratory of Medicinal Chemistry, Faculty of Pharmacy and Food Sciences and Institute of Biomedicine (IBUB), University of Barcelona, Av. de Joan XXIII, 27-31, 08028 Barcelona, Spain.

Table of Contents

1. General Information 2

2. Experimental Procedures 3

2.1. Preparation of Ugi Adducts 6 and 7 3

2.1.1. General Procedure A 3

2.1.2. General Procedure B 3

2.2. Preparation of Povarov Adducts 9 4

2.2.1. General Procedure C: MCR Povarov using activated alkenes as dienophiles 4

2.2.2. General Procedure D: MCR Povarov using phenylacetylenes as dienophiles 4

3. Theoretical Molecular Properties 5

4. Characterization Data 6

4.1. Ugi Adducts (6a-c, 7a-b) 6

4.2. Povarov Adducts (9a-i) 8

5. Copies of NMR spectra. 14

1. General Information

All chemicals were purchased from commercial sources and were used as received unless otherwise stated. All reactions were performed under argon in dried glassware, unless otherwise stated. Microwave-irradiated reactions were carried out using a Biotage Initiator Classic. Column chromatographies were carried out on commercial silica gel 60 (230–400 mesh ASTM) using hexane-ethyl acetate as eluent solvents. Automated Flash column chromatographies were carried out using an Isolera Prime Biotage equipped with dual UV detection over prepacked normal phase silica gel columns (4, 12 and 24 g) and using hexane-ethyl acetate as eluent solvents. Thin layer chromatographies were done using pre-coated Merk silica gel 60 F_{254} plates and visualized under UV light at 254 nm and 365 nm.

The ^{1}H NMR spectra were recorded on 400 MHz spectrometers. The ^{13}C NMR spectra were recorded at 100 MHz. The ^{19}F spectra were recorded at 376 MHz. Chemical shifts were reported in ppm(δ) as s (singlet), d (doublet), t (triplet), dd (doublet of doublet), m (multiplet), br s (broad singlet), etc. The residual solvent signals were used as references. Coupling constants (*J*) are reported in Hertz. ^{13}C NMR, and ^{19}F NMR were broadband decoupled from hydrogen nuclei. HPLC-MS spectra were carried out using Agilent 1260 Infinity II. The analysis was conducted on a Poroshell 120 EC-C15 (4.6 mm × 50 mm, 2.7 µm) at 40 °C with mobile phase A (H_2O + 0.05% formic acid) and B (ACN + 0.05% formic acid) using a gradient elution and flow rate 0.6 mL/min. The DAD detector was set at 254 or 220 nm, the injection volume was 5 µL, and oven temperature was 40 °C. The University of Barcelona Mass Spectrometry Service and. The Central Analysis Service of the UPV/EHU performed the High-Resolution Mass Spectrometry High-resolution mass spectra (HRMS) were obtained by the positive-ion electrospray ionization (ESI)method with a time-of-flight Q-TOF system.

2. Experimental Procedures

2.1. Preparation of Ugi Adducts 6 and 7

2.1.1. General Procedure A

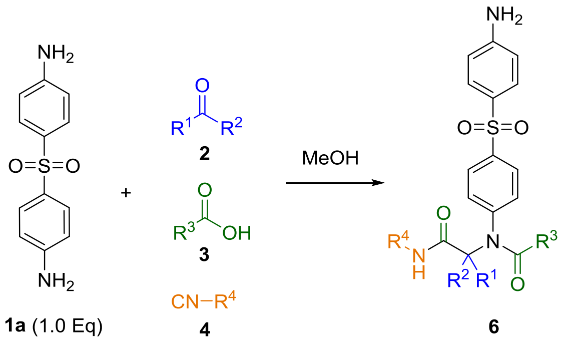

A mixture of dapsone **1a** (1.0 mmol, 1.0 eq.), carbonylic compound **2** (10.0 mmol, 10.0 eq.) and carboxylic acid **3** (1.0 mmol, 1.0 eq.) were added to a Schlenk flask with 2.0 mL of MeOH. The reaction mixture stirred at room temperature for 10 minutes. Then the suitable isocyanide **4** (1.0 mmol, 1.0 eq.) was added into the mixture and the reaction was left stirring overnight at room temperature. After reaction completion was determined by LC-MS or TLC, the reaction mixture was evaporated under reduced pressure. The reaction crude was diluted with EtOAc (20 mL) and was mixed with saturated aqueous solution of $NaHCO_3$. The aqueous phase was separated and extracted with EtOAc (2 x 20 mL). The combined organic phases were dried with $MgSO_4$, filtered and evaporated under reduced pressure. The pure product **6** was obtained through automated flash chromatography using hexane-ethyl acetate as eluent solvents.

2.1.2. General Procedure B

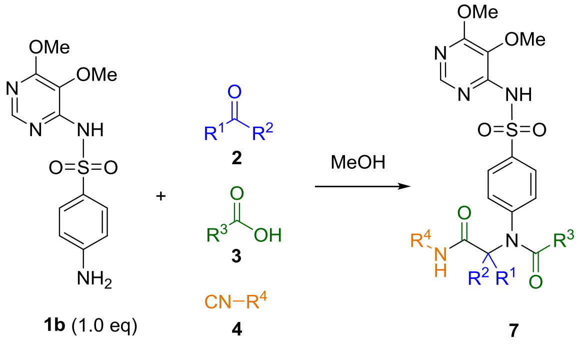

Sulfadoxine **1b** (1.0 mmol, 0.312 g, 1 eq.), aldehyde **2** (1.0 mmol, 1 eq.), and benzoic acid **3** (1.0 mmol, 0.122 g, 1.0 eq.) were dissolved in 2.0 mL of dry methanol and was left stirring 10 minutes at room temperature. Then, the isocyanide **4** (1.0 mmol, 1.0 eq.) was added into the mixture and the reaction was left stirring under nitrogen at room temperature until TLC analysis indicated the consumption of the starting materials. MeOH was evaporated under reduced pressure and the reaction crude was diluted with ethyl acetate (20.0 mL) and mixed with saturated aqueous solution of $NaHCO_3$. The organic and aqueous phase were separated and the aqueous phase was extracted with AcOEt (2x 20 mL). The combined organic phases were dried with $MgSO_4$, filtered and evaporated under reduced pressure. The corresponding products **7**

were purified by flash column chromatography on silica gel using hexane-ethyl acetate (70:30) as eluent solvents.

2.2. Preparation of Povarov Adducts 9

2.2.1. General Procedure C: MCR Povarov using activated alkenes as dienophiles

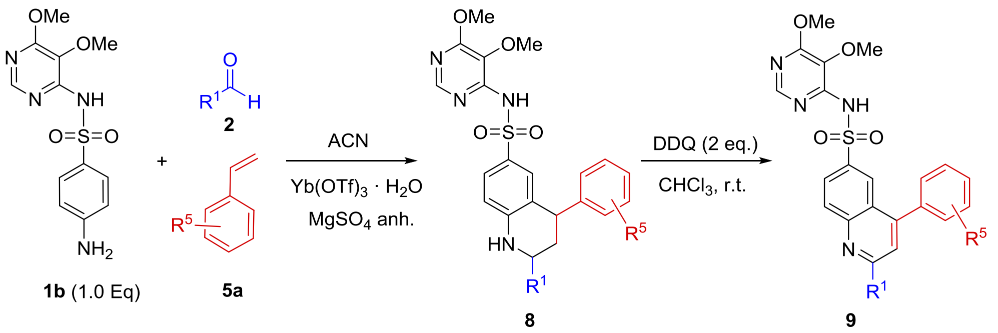

To a suspension of sulfadoxine **1b** (1.0 mmol, 1.0 eq.), aldehyde **2** (1.0 mmol, 1.0 eq.) and ytterbium triflate (%20 mmol) in 5 mL of dry acetonitrile, and in presence of anhydrous $MgSO_4$ (800 mg), the corresponding dienophile **5a** (1.5 mmol, 1.5 eq.) was added. The resulting mixture was stirred under nitrogen atmosphere at room temperature until TLC analysis indicated the disappearance of the starting materials. The solution was then diluted with dichloromethane (20 mL), washed with water (2 x 10 mL) and the aqueous layer was again extracted with dichloromethane (2 x 10 mL). The organic phase was dried over anhydrous $MgSO_4$, filtered and concentrated under vacuum. The corresponding crude residue, was dissolved in $CHCl_3$ (15 mL), and DDQ (2 mmol) was added to the solution. The reaction mixture was stirred overnight at room temperature in an open vessel. A saturated aqueous solution of $NaHCO_3$ (10 mL) was added, the resulting mixture was extracted with dichloromethane (2 x 25 mL), dried over anhydrous $MgSO_4$, filtered and concentrated under reduced pressure. The crude residue obtained was purified by flash column chromatography on silica gel (hexane-ethyl acetate 80:20) to afford the dehydrogenated compounds **9**.

2.2.2. General Procedure D: MCR Povarov using phenylacetylenes as dienophiles

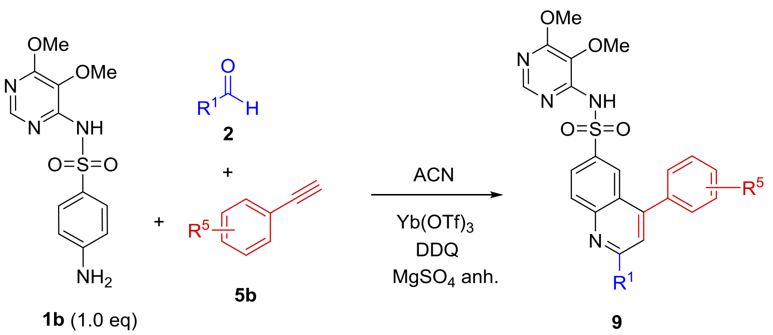

Sulfadoxine **1b** (0.483 mmol, 0.150 g, 1 eq.), aldehyde **2** (0.483 mmol, 1 eq.) and ytterbium triflate (0.097 mmol, 0.06 g, %20 mmol) were dissolved in 5 mL of dry acetonitrile, in presence of anhydrous $MgSO_4$ (400 mg). The corresponding phenyl acetylene **5b** (0.7245 mmol, 1.5 eq.) and DDQ (0.483 mmol, 0.109 g, 1 eq.) were added to the suspension at room temperature. The mixture was then stirred under nitrogen at the suitable temperature until TLC analysis indicated

the consumption of the starting materials. The contents were added to a saturated aqueous solution of $NaHCO_3$ (10 mL) and extracted with dichloromethane (2 x 10 mL). The organic phase was dried over anhydrous $MgSO_4$, filtered and concentrated under vacuum. Removing the solvent, the crude obtained was purified by column chromatography on silica gel using an elution gradient of hexane-ethyl acetate (80-20) to yield the pure products **9**.

3. Theoretical Molecular Properties

Molecular properties of the synthesized molecules **1a**, **1b**, **6**, **7**, **9** were calculated using SwissADME platform (http://www.swissadme.ch). Some of the main features have been summarized in Table S1. For the complete information see the full Excel file. In the Supplementary material.

Table S1. Selected data for the synthesized compounds (SwissADME)

Compound	Formula	MW	#Rotatable bonds	#H-bond acceptors	#H-bond donors	TPSA	Consensus Log P	Ali Class	GI absorption	BBB permeant	Lipinski #violations
1a	C12H12N2O2S	248,3	2	2	2	94,56	1,55	Soluble	High	No	0
1b	C12H14N4O4S	310,3	5	6	2	124,8	0,77	Soluble	High	No	0
6a	C31H29Cl2N3O4S	610,6	10	4	2	118	5,62	Poorly soluble	Low	No	2
6b	C27H30N4O4S	506,6	9	5	2	130,8	3,1	1oderately solub	Low	No	1
6c	C31H34F3N3O4S	601,7	11	7	2	118	5,41	Poorly soluble	Low	No	2
7a	C30H37N5O6S	595,7	13	8	2	148,2	3,69	Poorly soluble	Low	No	2
7b	C31H32ClN5O6S	638,1	13	8	2	148,2	4,03	Poorly soluble	Low	No	2
9a	C27H21FN4O4S	516,5	7	8	1	111,7	4,49	Poorly soluble	Low	No	1
9b	C28H24N4O4S	512,6	7	7	1	111,7	4,53	Poorly soluble	Low	No	1
9c	C28H21F3N4O4S	566,6	8	10	1	111,7	5,13	Poorly soluble	Low	No	1
9d	C28H24N4O5S	528,6	8	8	1	120,9	4,12	Poorly soluble	Low	No	1
9e	C29H24N4O6S	556,6	9	9	1	138	4,04	Poorly soluble	Low	No	1
9f	C27H20ClFN4O4S	551	7	8	1	111,7	4,97	Poorly soluble	Low	No	1
9g	C28H23ClN4O4S	547	7	7	1	111,7	5,08	Poorly soluble	Low	No	1
9h	C28H20ClF3N4O4S	601	8	10	1	111,7	5,68	Poorly soluble	Low	No	1
9i	C28H23ClN4O5S	563	8	8	1	120,9	4,68	Poorly soluble	Low	No	1

4. Characterization Data

4.1. Ugi Adducts (6a-c, 7a-b)

***N*-(4-((4-aminophenyl)sulfonyl)phenyl)-*N*-(2-(tert-butylamino)-1-(4-chlorophenyl)-2-oxoethyl)-4-chlorobenzamide (6a)**

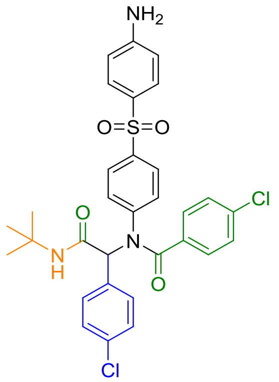

Following the *general procedure A,* compound **6a** (0.202 g, 33 %) was obtained as an off-white powder.

^{1}H NMR (400 MHz, $CDCl_3$) δ 7.63 – 7.43 (m, 4H), 7.19 – 7.02 (m, 10H), 6.72 – 6.61 (m, 2H), 6.01 (s, 1H), 5.56 (s, 1H), 4.19 (s, 2H), 1.34 (s, 9H).

^{13}C NMR (101 MHz, $CDCl_3$) δ 169.85, 168.01, 151.31, 144.80, 141.56, 136.39, 134.99, 133.62, 132.81, 131.46, 131.00, 130.12, 129.73, 129.01, 128.98, 128.33, 127.65, 114.22, 65.99, 52.14, 28.74.

HRMS: Calculated for $C_{31}H_{29}Cl_2N_3O_4S^+$, 610.1329 (M+H$^+$); found 610.1333.

2-(*N*-(4-((4-aminophenyl)sulfonyl)phenyl)acetamido)-*N*-cyclohexyl-2-(pyridin-4-yl)acetamide (6b)

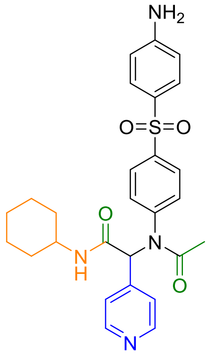

Following the *general procedure A*, compound **6b** (0.065 g, 25 %) was obtained as a yellow powder.

^{1}H NMR (400 MHz, $CDCl_3$) δ 8.41 (d, *J* = 5.6 Hz, 2H), 7.76 (d, *J* = 8.9 Hz, 2H), 7.64 (d, *J* = 8.7 Hz, 2H), 7.28 (s, 1H), 7.04 (d, *J* = 6.1 Hz, 2H), 6.67 (d, *J* = 8.7 Hz, 2H), 5.86 (s, 1H), 5.70 (d, *J* = 8.1 Hz, 1H), 4.20 (s, 2H), 3.85 – 3.72 (m, 1H), 1.93 (d, *J* = 13.2 Hz, 1H), 1.86 (s, 3H), 1.69 – 1.55 (m, 5H), 1.37 – 1.30 (m, 2H), 1.16 – 1.01 (m, 3H), 0.92 – 0.82 (m, 1H).

^{13}C NMR (101 MHz, $CDCl_3$) δ 170.94, 167.26, 151.58, 150.01, 144.16, 143.40, 143.27, 130.96, 130.08, 128.49, 128.28, 124.71, 114.33, 64.28, 49.17, 32.83, 32.79, 25.49, 24.86, 24.79, 23.44.

HRMS: Calculated for $C_{27}H_{31}N_4O_4S^+$, 507.2061 (M+H$^+$); found 507.2057.

1-(*N*-(4-((4-aminophenyl)sulfonyl)phenyl)-2-(4-(trifluoromethyl)phenyl)acetamido) -*N*-(*tert*-butyl)cyclopentane-1-carboxamide (6c)

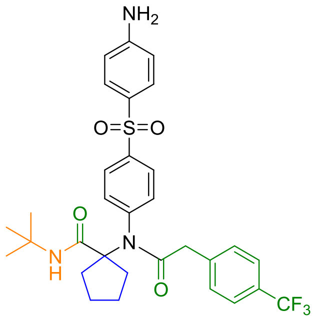

Following the *general procedure A*, compound **6c** (0.060 g, 21 %) was obtained as a white powder.

^{1}H NMR (400 MHz, $CDCl_3$) δ 7.84 (d, J = 8.6 Hz, 2H), 7.73 (d, J = 8.7 Hz, 2H), 7.34 (d, J = 8.5 Hz, 4H), 6.94 (d, J = 7.9 Hz, 2H), 6.70 (d, J = 8.7 Hz, 2H), 6.30 (s, 1H), 4.25 (s, 2H), 3.31 (s, 2H), 2.26 – 2.15 (m, 2H), 1.62 – 1.53 (m, 6H), 1.34 (s, 9H).

^{13}C NMR (101 MHz, $CDCl_3$) δ 172.46, 170.77, 151.58, 144.45, 143.53, 138.77, 131.12, 130.27, 129.53 (q, J_{C-F} = 32.6 Hz), 129.22, 128.68, 128.18, 125.38 (q, J_{C-F} = 4.0 Hz), 124.30 (q, J_{C-F} = 270.8 Hz), 114.41, 74.73, 51.32, 43.12, 36.98, 28.67, 22.88.

HRMS: Calculated for $C_{31}H_{35}F_3N_3O_4S^+$, 602.2295 (M+H$^+$); found 602.2298.

***N*-(1-(cyclohexylamino)-3-methyl-1-oxobutan-2-yl)-*N*-(4-(*N*-(5,6-dimethoxypyrimidin-4-yl)sulfamoyl)phenyl)benzamide (7a)**

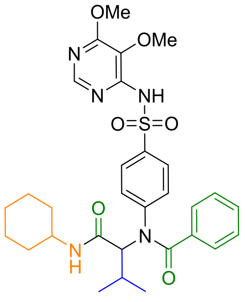

The general procedure B was followed using isobutyraldehyde (0.09 ml) and cyclohexyl isocyanide (0.124 ml) at room temperature for 48 h, affording 0.233 g (39 %) of the compound **7a** as a white solid.

^{1}H-NMR (400 MHz, $CDCl_3$) δ 8.06 (s, 1H), 7.92 (d, J = 8.9 Hz, 2H), 7.77 (s, 1H), 7.31 (d, J = 8.4 Hz, 2H), 7.24 – 7.18 (m, 1H), 7.14 – 7.05 (m, 4H), 6.89 (s, 1H), 4.51 (d, J = 11.2 Hz, 1H), 4.00 (s, 3H), 3.84 (s, 3H), 3.82 – 3.66 (m, 1H), 2.47 (s, 1H), 1.94 – 1.78 (m, 2H), 1.75 – 1.61 (m, 2H), 1.62 – 1.52 (m, 1H), 1.42 – 1.28 (m, 2H), 1.25 – 1.12 (m, 3H), 1.01 (d, J = 6.5 Hz, 3H), 0.94 (d, J = 6.5 Hz, 1H).

^{13}C-NMR (100 MHz, $CDCl_3$) δ 172.2, 169.3, 161.0, 151.0, 149.5, 146.7, 137.5, 135.6, 130.4, 129.2, 128.8, 128.5, 128.2, 126.6, 70.9, 60.7, 54.3, 48.2, 33.0, 32.9, 26.9, 25.6, 24.8, 24.7, 20.0, 19.9.

HRMS: Calculated for $C_{30}H_{37}N_5O_6S$, 595.2465 (M+H$^+$), found 595.2475.

***N*-(2-(*tert*-butylamino)-1-(4-chlorophenyl)-2-oxoethyl)-*N*-(4-(*N*-(5,6-dimethoxypyrimidin-4-yl)sulfamoyl)phenyl)benzamide (7b)**

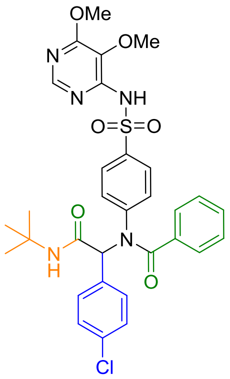

The *general procedure B* was followed using 4-chlorobenzaldehyde (0.141 g) and *tert*-butyl isocyanide (0.12 ml) at room temperature for 24 h, affording 0.380 g (53 %) of the compound **9b** as a white solid.

^{1}H-NMR (400 MHz, $CDCl_3$) δ 8.03 (s, 1H), 7.76 (d, *J* = 8.9 Hz, 2H), 7.68 (s, 1H), 7.24 - 6.95 (m, 11H), 6.12 (s, 1H), 5.65 (s, 1H), 4.02 (s, 3H), 3.82 (s, 3H), 1.36 (s, 9H).

^{13}C-NMR (100 MHz, $CDCl_3$) δ 171.1, 168.2, 161.0, 151.0, 149.4, 145.9, 137.5, 135.2, 134.9, 132.9, 131.4, 130.5, 130.2, 129.0, 128.9, 128.7, 128.0, 126.5, 65.7, 60.7, 54.4, 52.1, 28.8.

HRMS: Calculated for $C_{31}H_{32}ClN_5O_6S$, 637.1762 (M+H$^+$); found 637.1786.

4.2. Povarov Adducts (9a-i)

***N*-(5,6-dimethoxypyrimidin-4-yl)-2-(4-fluorophenyl)-4-phenylquinoline-6 sulfonamide (9a)**

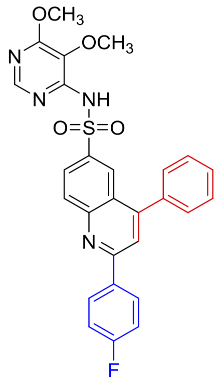

Following the *general procedure C*, the crude residue (0.198 g, 0.346 mmol) and DDQ (0.157 g, 0.692 mmol) were stirred and compound **9a** (0.047 g, 19 %) was obtained as a white solid. Compound **9a** could also be obtained following the general procedure E using 4-

fluorobenzaldehyde (0.052 ml) and ethynylbenzene (0.080 ml) as dienophile, heating to reflux for 72 h, yielding 0.055 g of compound **9a** in a 24 % yield.

^{1}H-NMR (400 MHz, $CDCl_3$) δ 8.86 (d, *J* = 2.1 Hz, 1H), 8.35 (dd, *J* = 9.0 Hz, *J* = 2.1 Hz, 1H), 8.29 (d, *J* = 9.2 Hz, 1H), 8.27 – 8.19 (m, 2H), 8.07 (s, 1H), 7.88 (s, 1H), 7.79 (s, 1H), 7.62 – 7.54 (m, 5H), 7.22 (t, *J* = 8.7 Hz, 2H), 3.97 (s, 3H), 3.85 (s, 3H).

^{13}C-NMR (100 MHz, $CDCl_3$) δ 164.4 (d, $J_{C\text{-}F}$ = 250.6 Hz), 161.0, 158.6, 151.1, 150.5, 149.7, 137.3, 136.7, 135.0 (d, $J_{C\text{-}F}$ = 3.2 Hz), 131.2, 129.9 (d, $J_{C\text{-}F}$ = 8.6 Hz), 129.6, 129.4, 129.2, 129.0, 127.2, 126.6, 124.8, 120.3, 116.2 (d, $J_{C\text{-}F}$ = 21.8 Hz), 60.7, 54.3.

^{19}F-NMR (376 MHz, $CDCl_3$) δ -111.14 – -111.25 ppm (m).

HRMS: Calculated for $C_{27}H_{21}FN_4O_4S$, 516.1268 (M+H$^+$), found 516.1278.

***N*-(5,6-dimethoxypyrimidin-4-yl)-4-phenyl-2-(*p*-tolyl)quinoline-6-sulfonamide (9b)**

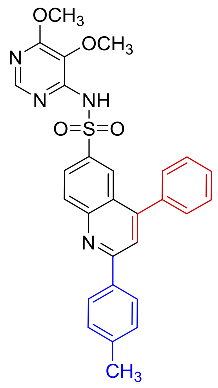

Following the *general procedure C*, the crude residue (0.344 g, 0.671 mmol) and DDQ (0.305 g, 1.34 mmol) were stirred. Compound **9b** (0.078 g, 19 %) was obtained as a white solid.

^{1}H-NMR (400 MHz, $CDCl_3$) δ 8.85 (d, *J* = 2.0 Hz, 1H), 8.33 (dd, *J* = 9.0 Hz, *J* = 2.0 Hz, 1H), 8.29 (d, *J* = 8.9 Hz, 1H), 8.12 (d, *J* = 8.2 Hz, 2H), 8.07 (s, 1H), 7.91 (s, 1H), 7.82 (s, 1H), 7.61 – 7.53 (m, 5H), 7.34 (d, *J* = 8.0 Hz, 2H), 3.97 (s, 3H), 3.84 (s, 3H), 2.44 (s, 3H).

^{13}C-NMR (100 MHz, $CDCl_3$) δ= 161.0, 159.7, 151.1, 150.8, 150.6, 149.7, 140.7, 137.4, 136.4, 136.04, 131.1, 129.9, 129.6, 129.3, 129.1, 128.9, 127.8, 127.1, 126.6, 124.8, 120.5, 60.7, 54.3, 21.6.

HRMS: Calculated for $C_{28}H_{24}N_4O_4S$, (M+H$^+$) 512.1518, found 512.1522.

N-(5,6-dimethoxypyrimidin-4-yl)-4-phenyl-2-(4-(trifluoromethyl)phenyl)quinoline-6-sulfonamide (9c)

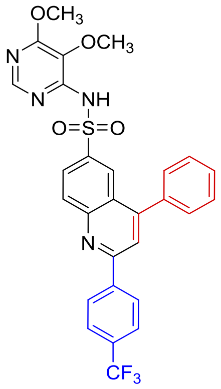

Following the *general procedure C*, the crude residue (0.257 g, 0.511 mmol) and DDQ (0.232 g, 1.022 mmol) were stirred and compound **9c** was obtained as a white solid (0.114 g, 42 %). Compound **9c** could also be obtained using the general procedure E, using 4-trifluorobenzaldehyde (0.068 ml) and ethynylbenzene as dienophile (0.080 ml) heating to reflux for 24 h, yielding 0.097 g of compound **9c** in a 35 % yield.

^{1}H-NMR (400 MHz, $CDCl_3$) δ 8.90 (d, *J* = 2.1 Hz, 1H), 8.38 (dd, *J* = 9.0 Hz, *J* = 2.1 Hz, 1H), 8.36 – 8.31 (m, 3H), 8.07 (s, 1H), 7.94 (s, 1H), 7.84 (s, 1H), 7.80 (d, *J* = 8.3 Hz, 2H), 7.64 – 7.55 (m, 5H), 3.97 (s, 3H), 3.86 (s, 3H).

^{13}C-NMR (100 MHz, $CDCl_3$) δ 161.0, 158.1, 151.4, 151.0, 150.5, 149.6, 142.2, 137.1, 132.0 (d, J_{C-F} = 32.5 Hz), 131.4, 129.6, 129.5, 129.2, 129.0, 128.2, 127.4, 126.7, 126.0 (q, J_{C-F} = 3.8 Hz), 125.2, 124.2 (d, J_{C-F} = 272.4 Hz), 120.5, 60.7, 54.3.

^{19}F-NMR (376 MHz, $CDCl_3$) δ= -62.69.

HRMS: Calculated for $C_{28}H_{21}F_3N_4O_4S$, 566.1236 ($M+H^+$); found 566.1240.

N-(5,6-dimethoxypyrimidin-4-yl)-2-(4-methoxyphenyl)-4-phenylquinoline-6-sulfonamide (9d)

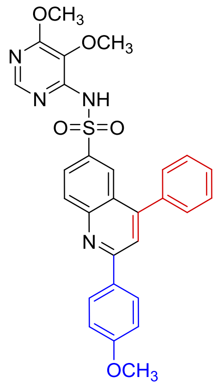

Following the *general procedure C*, sulfadoxine (0.250 g, 0.8 mmol), 4-methoxybenzaldehyde (0.098 ml, 0.8 mmol), styrene as dienophile (0.14 ml, 1.2 mmol) and ytterbium triflate (0.100 g, 0.16 mmol) were stirred and heated to reflux for 48 h. The dehydrogenated product **9d** was directly obtained (0.072 g, 17 %) by flash column chromatography on silica gel (hexane-ethyl acetate 80:20).

^{1}H-NMR (400 MHz, $CDCl_3$) δ= 8.83 (d, *J* = 2.0 Hz, 1H), 8.32 (dd, *J* = 8.9 Hz, *J* = 2.1 Hz, 1H), 8.27 (d, *J* = 9.1 Hz, 1H), 8.20 (d, *J* = 8.9 Hz, 2H), 8.07 (s, 1H), 7.88 (s, 1H), 7.78 (s, 1H), 7.61 – 7.54 (m, 5H), 7.05 (d, *J* = 8.9 Hz, 2H), 3.97 (s, 3H), 3.90 (s, 3H), 3.85 (s, 3H).

^{13}C-NMR (100 MHz, $CDCl_3$) δ 161.7, 161.0, 159.2, 151.1, 150.7, 149.7, 137.5, 136.1, 130.9, 129.6, 129.4, 129.2, 129.1, 128.9, 127.1, 126.6, 124.6, 120.2, 114.5, 60.7, 55.6, 54.3.

HRMS: Calculated for $C_{28}H_{24}N_4O_5S$, ($M+H^+$) 528.1467; found 528.1467.

Methyl 4-(6-(*N*-(5,6-dimethoxypyrimidin-4-yl)sulfamoyl)-4-phenylquinolin-2-yl)benzoate (9e)

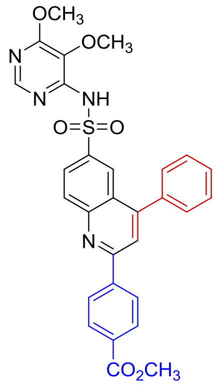

Following the *general procedure C*, the crude residue (0.412 g, 0.735 mmol) and DDQ (0.333 g, 1.47 mmol) were stirred. Compound **9e** (0.166 g, 37 %) was obtained as a white solid.

^{1}H-NMR (400 MHz, $CDCl_3$) δ 8.89 (d, *J* = 2.0 Hz, 1H), 8.37 (dd, *J* = 9.0 Hz, *J* =2.0 Hz, 1H), 8.33 (dd, *J* = 9.0 Hz, *J* =0.7 Hz, 1H), 8.30 (d, *J* = 8.7 Hz, 2H), 8.20 (d, *J* = 8.7 Hz, 2H), 8.07 (s, 1H), 7.96 (s, 1H), 7.82 (s, 1H), 7.63 – 7.52 (m, 5H), 3.97 (s, 3H), 3.97 (s, 3H), 3.85 (s, 3H).

^{13}C-NMR (100 MHz, $CDCl_3$) δ 166.9, 161.0, 158.5, 151.3, 151.1, 150.5, 149.6, 142.9, 137.1, 131.5, 131.4, 130.3, 129.6, 129.4, 129.2, 129.0, 127.9, 127.3, 126.7, 125.2, 120.7, 60.7, 54.3, 52.5.

HRMS: Calculated for $C_{29}H_{24}N_4O_6S$, ($M+H^+$) 556.1417; found 556.1423.

2-(4-Chlorophenyl)-*N*-(5,6-dimethoxypyrimidin-4-yl)-4-(4-fluorophenyl)quinoline-6-sulfonamide (9f)

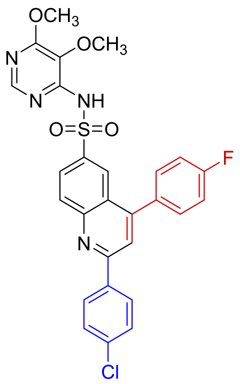

The *general procedure D* was followed using 4-chlorobenzaldehyde (0.068 g) and 1-ethynyl-4-fluorobenzene (0.087 ml), heated to reflux and stirring for 24 h to afford 0.075 g (28 %) of the compound **9f** as a white solid.

^{1}H-NMR (400 MHz, $CDCl_3$) δ= 8.80 (d, *J* = 2.0 Hz, 1H), 8.36 (dd, *J* = 8.9 Hz, *J* = 2.1 Hz, 1H), 8.29 (d, *J* = 8.9 Hz, 1H), 8.17 (d, *J* = 8.6 Hz, 2H), 8.07 (s, 1H), 7.86 (s, 1H), 7.84 (s, 1H), 7.57-7.50 (m, 4H), 7.31 (t, *J* = 8.5 Hz, 2H), 3.97 (s, 3H), 3.86 (s, 3H),

^{13}C-NMR (100 MHz, $CDCl_3$) δ 163.5 (d, $J_{C\text{-}F}$ = 246.5 Hz), 161.0, 158.4, 151.0, 150.5, 150.0, 149.6, 137.1, 137.0, 136.8, 133.2 (d, $J_{C\text{-}F}$ = 3.4 Hz), 131.5, 131.3 (d, $J_{C\text{-}F}$ = 5.3 Hz), 129.4, 129.2, 128.6, 127.4, 126.7, 124.9, 120.2, 116.4 (d, $J_{C\text{-}F}$ = 21.7 Hz), 60.7, 54.4.

^{19}F-NMR (376 MHz, $CDCl_3$) δ -111.76 ppm.

HRMS: Calculated for $C_{27}H_{20}ClFN_4O_4S$, 550.0900 (M+H$^+$); found 550.0878.

2-(4-Chlorophenyl)-*N*-(5,6-dimethoxypyrimidin-4-yl)-4-(*p*-tolyl)quinoline-6-sulfonamide (9g)

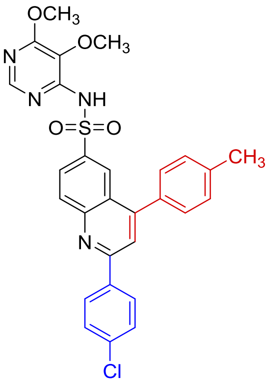

The *general procedure D* was followed using 4-chlorobenzaldehyde (0.068 g) and 1-ethynyl-4-methyl-benzene (0.092 ml), heated to reflux and stirring for 24 h to afford 0.140 g (53 %) of the compound **9g** as a white solid.

^{1}H-NMR (400 MHz, $CDCl_3$) δ 8.87 (d, *J* = 2.1 Hz, 1H), 8.34 (dd, *J* = 8.9 Hz, *J* =2.1 Hz, 1H), 8.28 (d, *J* = 8.9 Hz, 1H), 8.17 (d, *J* = 8.7 Hz, 2H), 8.09 (s, 1H), 7.87 (s, 1H), 7.80 (s, 1H), 7.51 (d, *J* = 8.7 Hz, 2H), 7.46 – 7.39 (m, 4H), 3.97 (s, 3H), 3.85 (s, 3H), 2.51 (s, 3H).

^{13}C-NMR (100 MHz, $CDCl_3$) δ 161.0, 158.4, 151.3, 151.1, 150.6, 149.7, 139.5, 137.3, 136.6, 136.6, 134.3, 131.2, 129.9, 129.5, 129.3, 129.2, 127.2, 126.7, 125.1, 120.1, 60.7, 54.3, 21.5.

HRMS: Calculated for $C_{28}H_{23}ClN_4O_4S$, 546.1129 (M+H$^+$); found 546.1131.

2-(4-Chlorophenyl)-*N*-(5,6-dimethoxypyrimidin-4-yl)-4-(4-(trifluoromethyl)phenyl)quinoline-6-sulfonamide (9h)

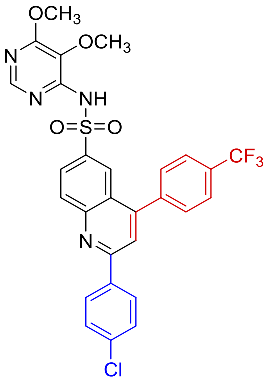

The *general procedure D* was followed using 4-chlorobenzaldehyde (0.068 g) and 1-ethynyl-4-(trifluoromethyl)benzene (0.118 ml) heated to reflux and stirring for 72 h to afford 0.022g (8 %) of compound **9h** as a white solid.

^{1}H-NMR (400 MHz, $CDCl_3$) δ 8.74 (d, *J* = 2.1 Hz, 1H), 8.38 (dd, *J* = 9.0, *J* = 2.1 Hz, 1H), 8.32 (d, *J* = 9.0 Hz, 1H), 8.17 (d, *J* = 8.6 Hz, 2H), 8.06 (s, 1H), 7.89 (s, 1H), 7.88 (d, *J* = 7.9 Hz, 2H), 7.83 (s, 1H), 7.69 (d, *J* = 8.1 Hz, 2H), 7.52 (d, *J* = 8.6 Hz, 2H), 3.97 (s, 3H), 3.87 (s, 3H).

^{13}C-NMR (100 MHz, $CDCl_3$) δ 161.0, 158.4, 151.0, 150.4, 149.6, 149.5, 140.8, 137.3, 136.9, 136.9, 131.6 (d, $J_{C\text{-}F}$ = 32.2 Hz), 131.4, 130.0, 129.4, 129.2, 128.2, 127.6, 126.7, 126.2 (q, $J_{C\text{-}F}$ = 3.6 Hz), 124.5, 124.0 (d, $J_{C\text{-}F}$ = 272.4 Hz), 120.2, 60.8, 54.4.

^{19}F-NMR (376 MHz, $CDCl_3$) δ= -62.63

HRMS: Calculated for $C_{28}H_{20}ClF_3N_4O_4S$, 600.0846 (M+H^{+}); found 600.0847.

2-(4-Chlorophenyl)-*N*-(5,6-dimethoxypyrimidin-4-yl)-4-(4-methoxyphenyl)quinoline-6-sulfonamide (9i)

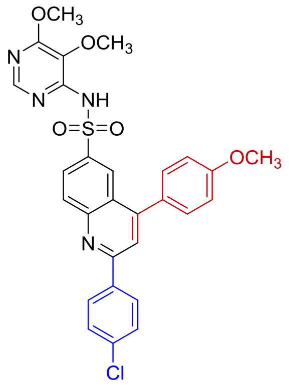

The *general procedure D* was followed using 4-chlorobenzaldehyde (0.068 g) and 1-ethynyl-4-methoxy-benzene (0.094 ml), at room temperature and stirring for 16 h to afford 0.101 g (37 %) of the compound **9i** as a yellow solid.

^{1}H-NMR (400 MHz, $CDCl_3$) δ 8.89 (d, *J*= 2.1 Hz, 1H), 8.34 (dd, *J* = 9.0 Hz, *J*= 2.1 Hz, 1H), 8.27 (dd, *J* = 8.9 Hz, *J* = 0.6 Hz, 1H), 8.17 (d, *J* = 8.6 Hz, 2H), 8.09 (s, 1H), 7.86 (s, 1H), 7.81 (s, 1H), 7.54 – 7.46 (m, 4H), 7.13 (d, *J* = 8.7 Hz, 2H), 3.97 (s, 3H), 3.94 (s, 3H), 3.86 (s, 3H).

^{13}C-NMR (100 MHz, $CDCl_3$) δ 161.0, 160.6, 158.3, 151.0, 150.9, 150.6, 149.7, 137.3, 136.6, 136.5, 131.2, 130.9, 129.4, 129.3, 129.1, 129.0, 127.1, 126.7, 125.1, 120.1, 114.7, 60.7, 55.6, 54.3.

HRMS: Calculated for $C_{28}H_{23}ClN_4O_5S$, 562.1078 (M+H^{+}); found 562.1096.

5. Copies of NMR spectra.

***N*-(4-((4-aminophenyl)sulfonyl)phenyl)-*N*-(2-(tert-butylamino)-1-(4-chlorophenyl)-2-oxoethyl)-4-chlorobenzamide (6a)**

^{1}H-NMR(400MHz,$CDCl_3$)

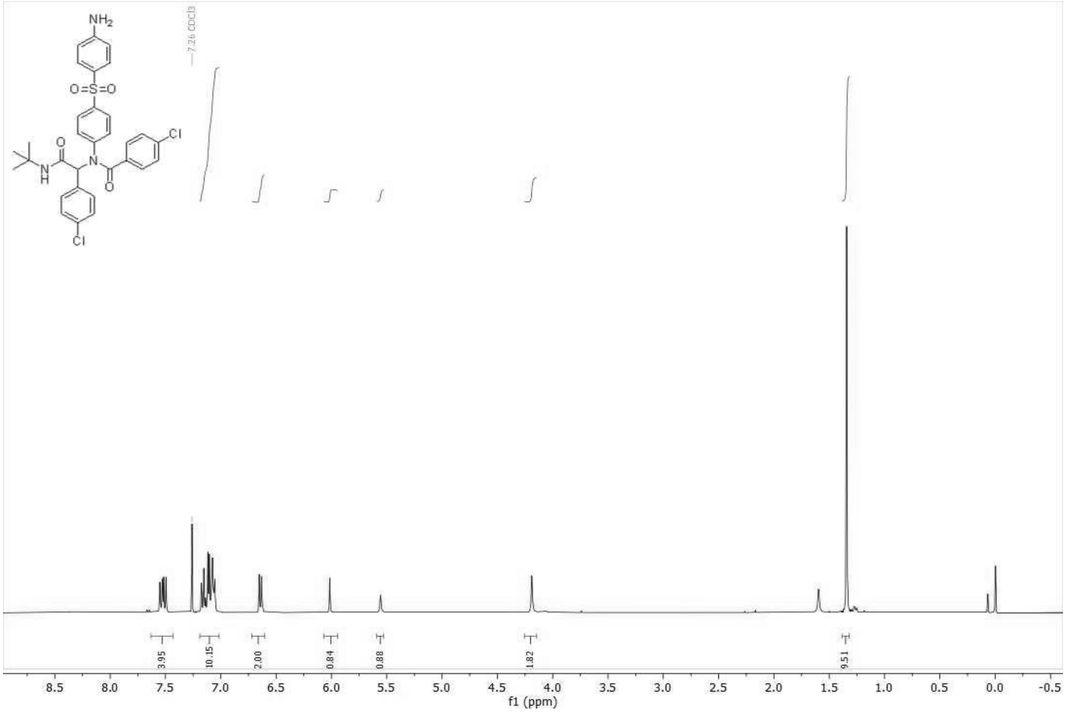

^{13}C-NMR (101 MHz, $CDCl_3$)

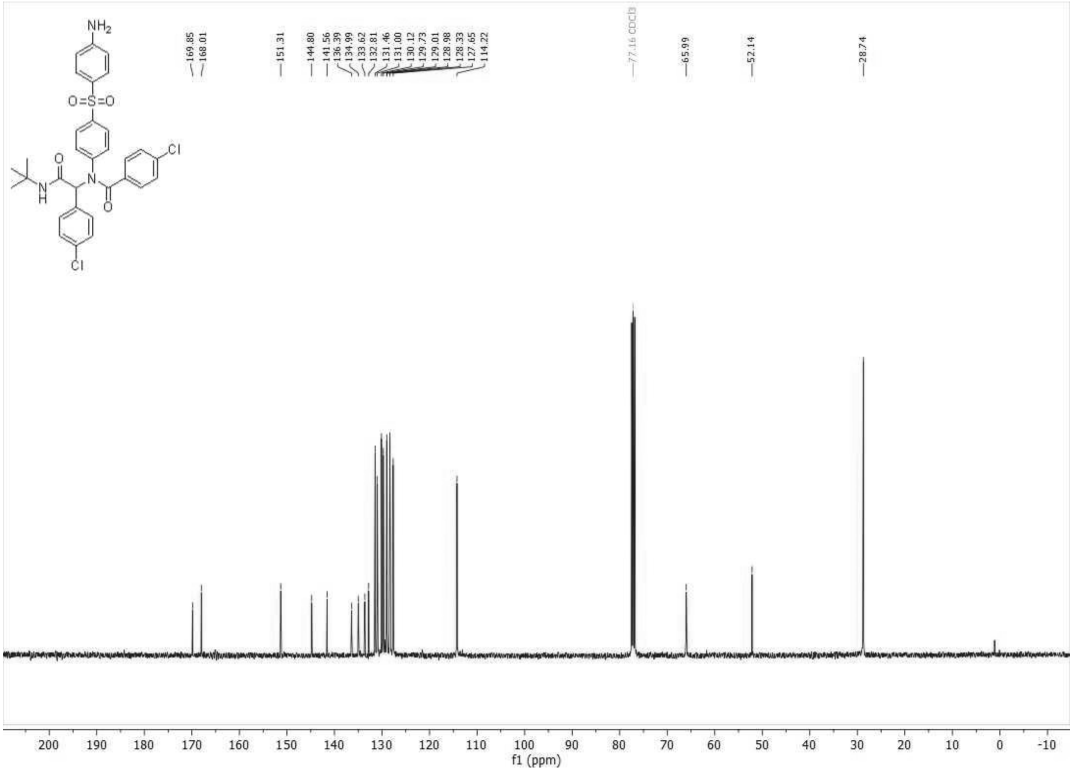

2-(*N*-(4-((4-aminophenyl)sulfonyl)phenyl)acetamido)-*N*-cyclohexyl-2-(pyridin-4-yl)acetamide (6b)

^{1}H-NMR(400MHz,$CDCl_3$)

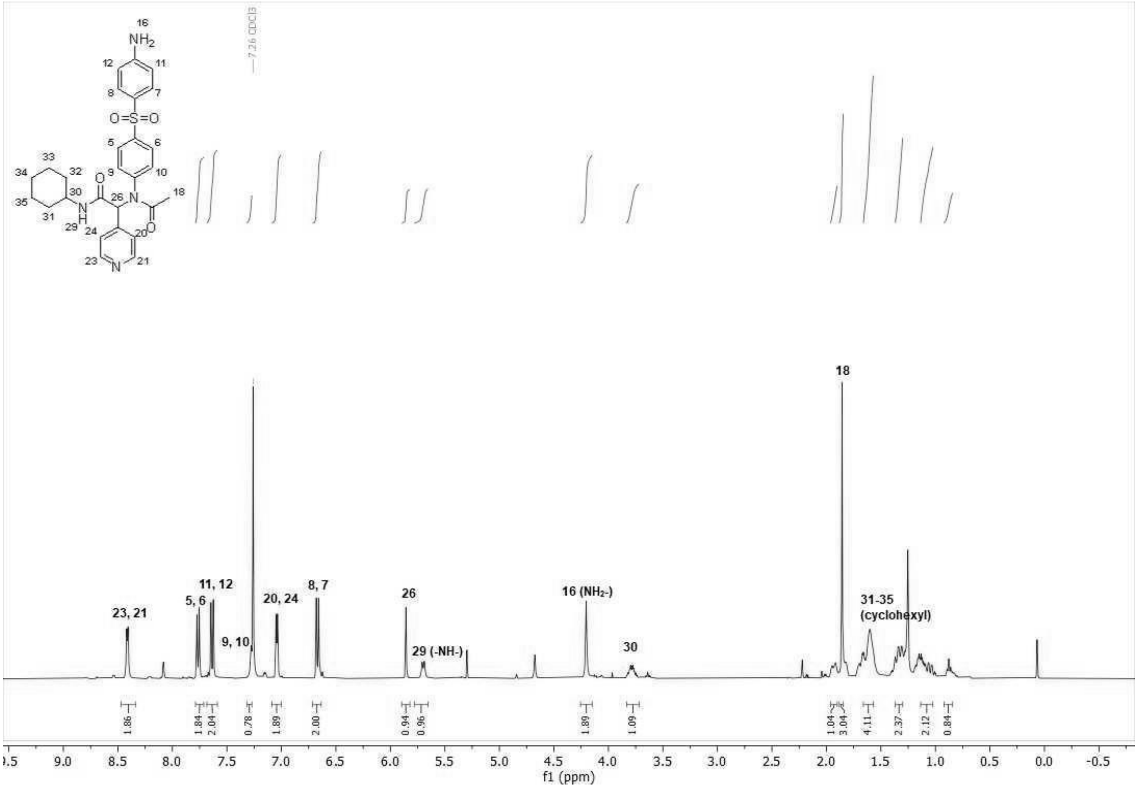

^{1}H-^{1}H COSY

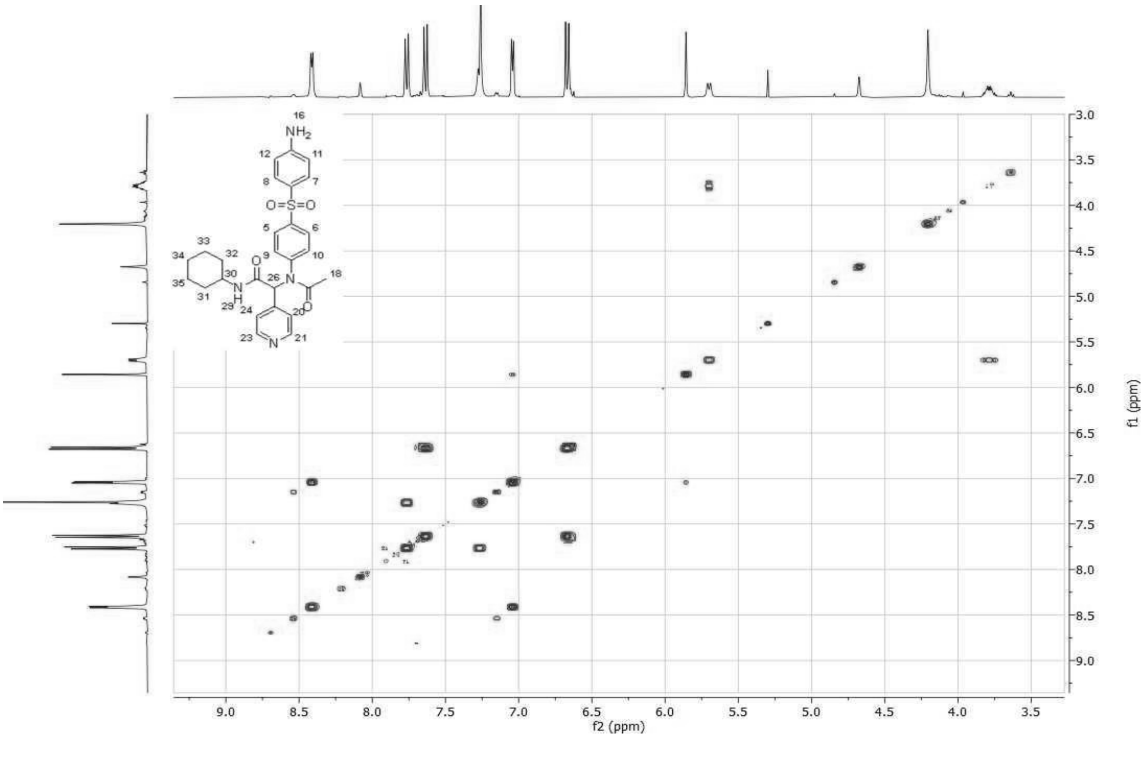

^{13}C-NMR (101 MHz, $CDCl_3$)

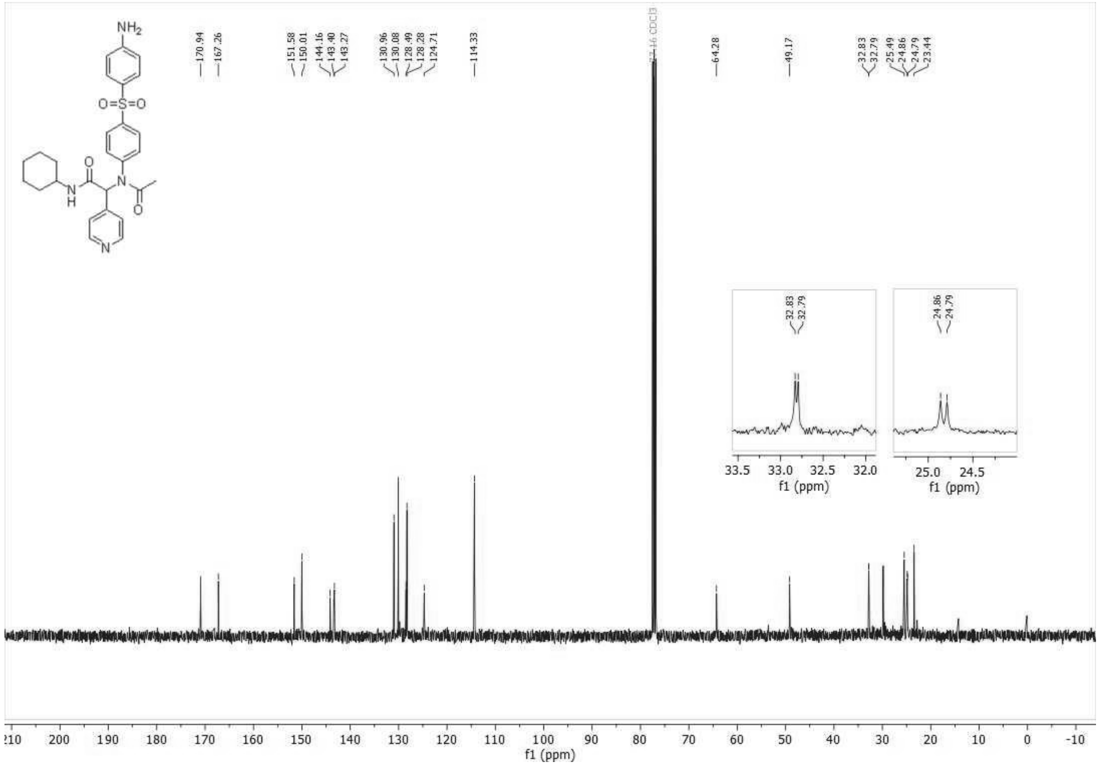

1-(*N*-(4-((4-aminophenyl)sulfonyl)phenyl)-2-(4-(trifluoromethyl)phenyl)acetamido)-*N*-(tert-butyl)cyclopentane-1-carboxamide (6c)

^{1}H-NMR(400MHz,$CDCl_3$)

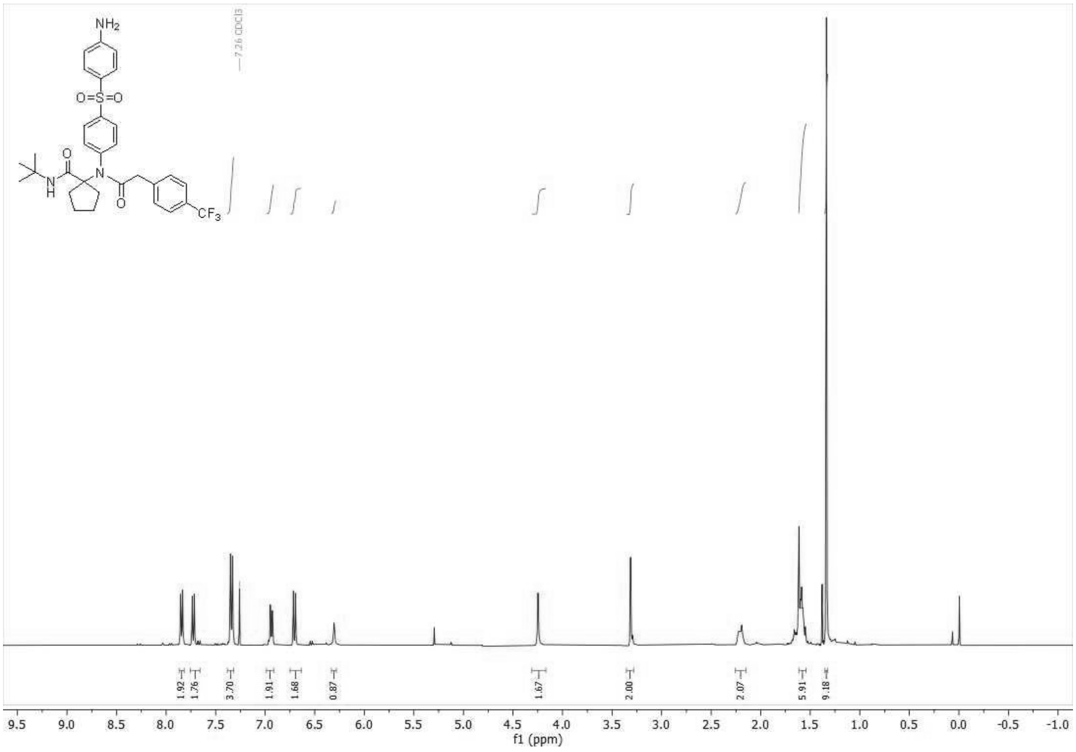

^{13}C-NMR (101 MHz, $CDCl_3$)

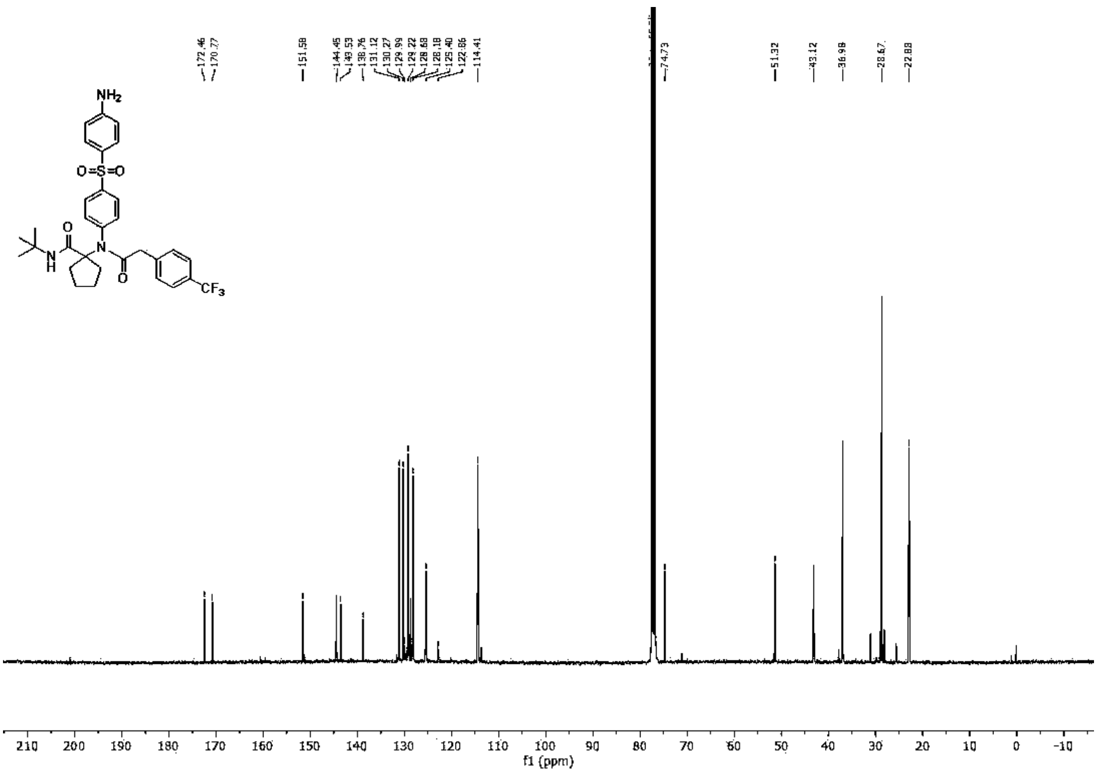

***N*-(1-(cyclohexylamino)-3-methyl-1-oxobutan-2-yl)-*N*-(4-(*N*-(5,6-dimethoxypyrimidin-4-yl)sulfamoyl)phenyl)benzamide(7a)**

^{1}H-NMR(400MHz,$CDCl_3$)

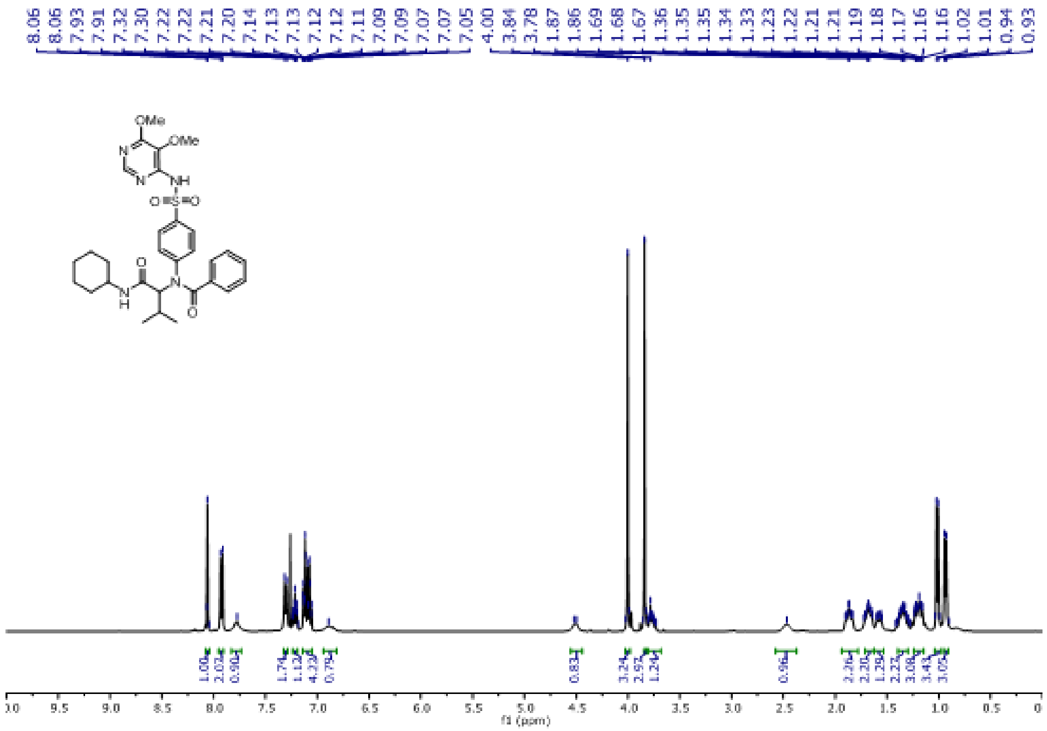

^{13}C-NMR (100 MHz, $CDCl_3$)

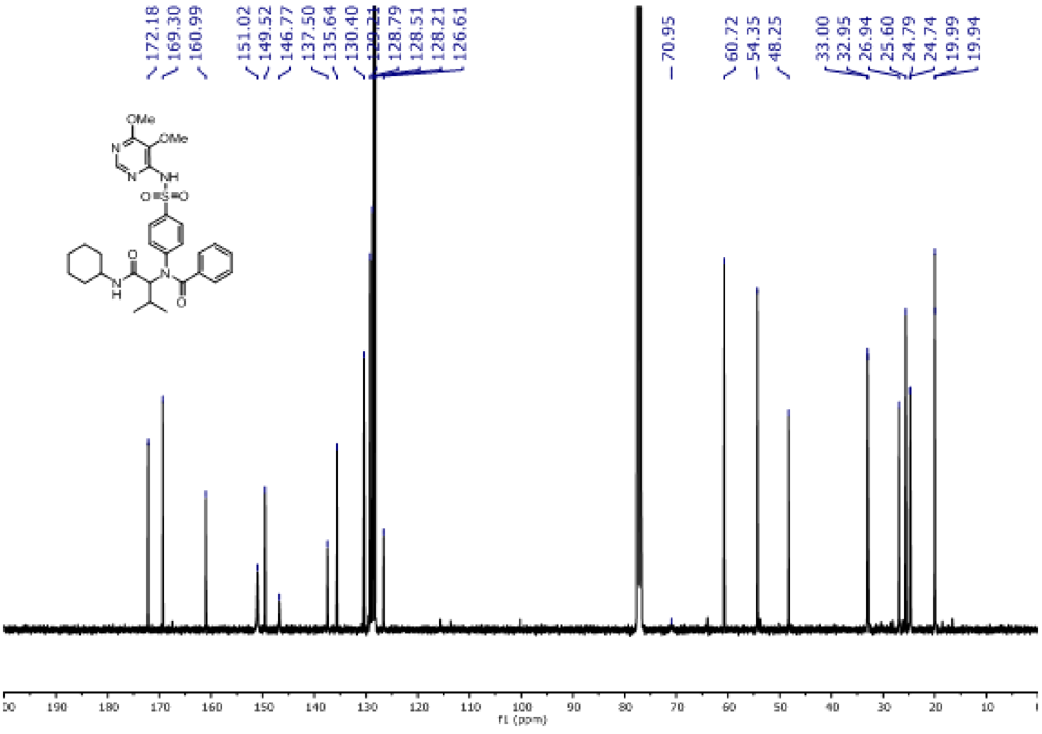

^{1}H-^{13}C HSQC

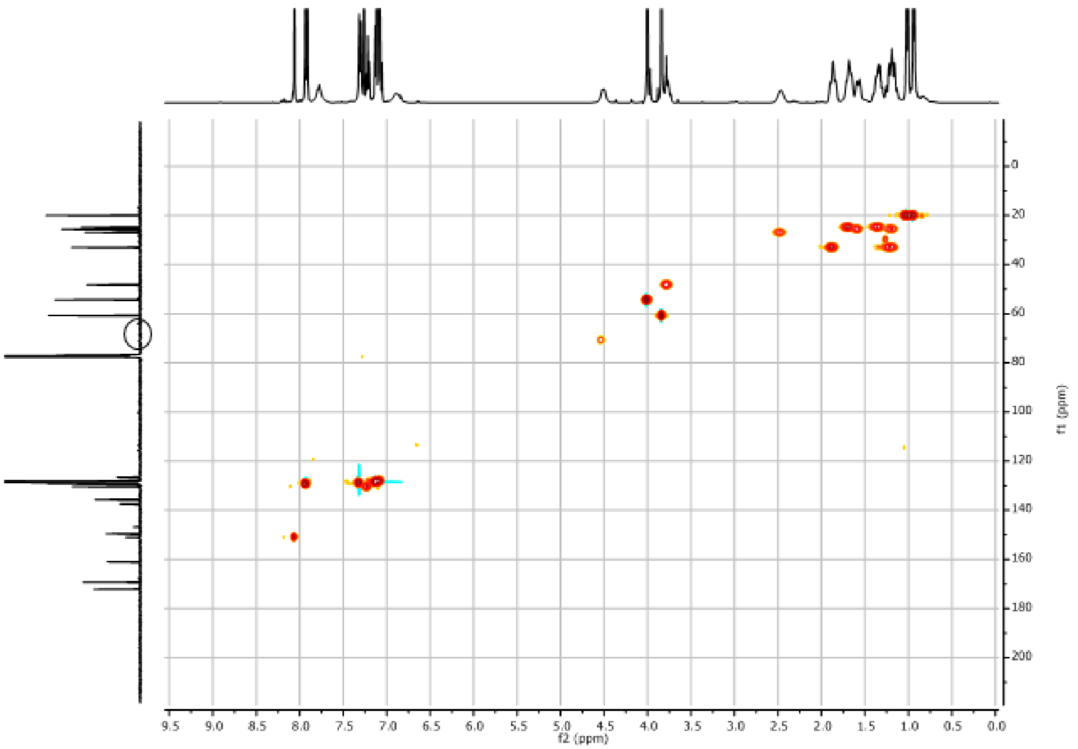

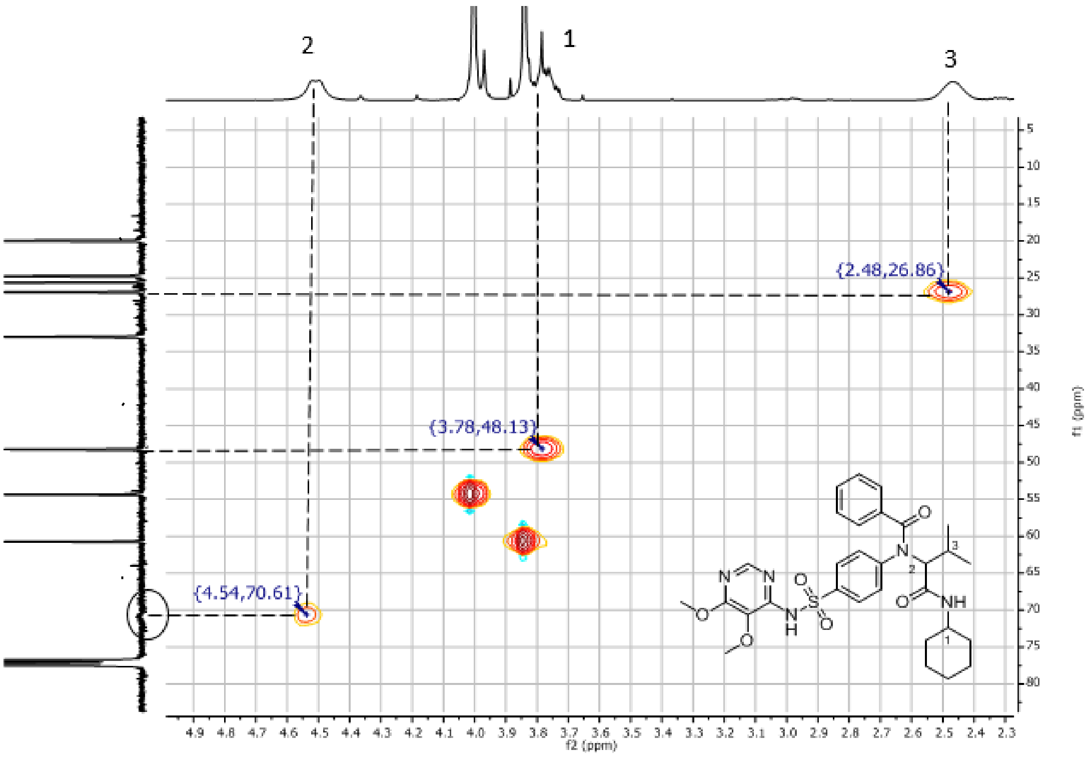

^{1}H-^{1}H COSY

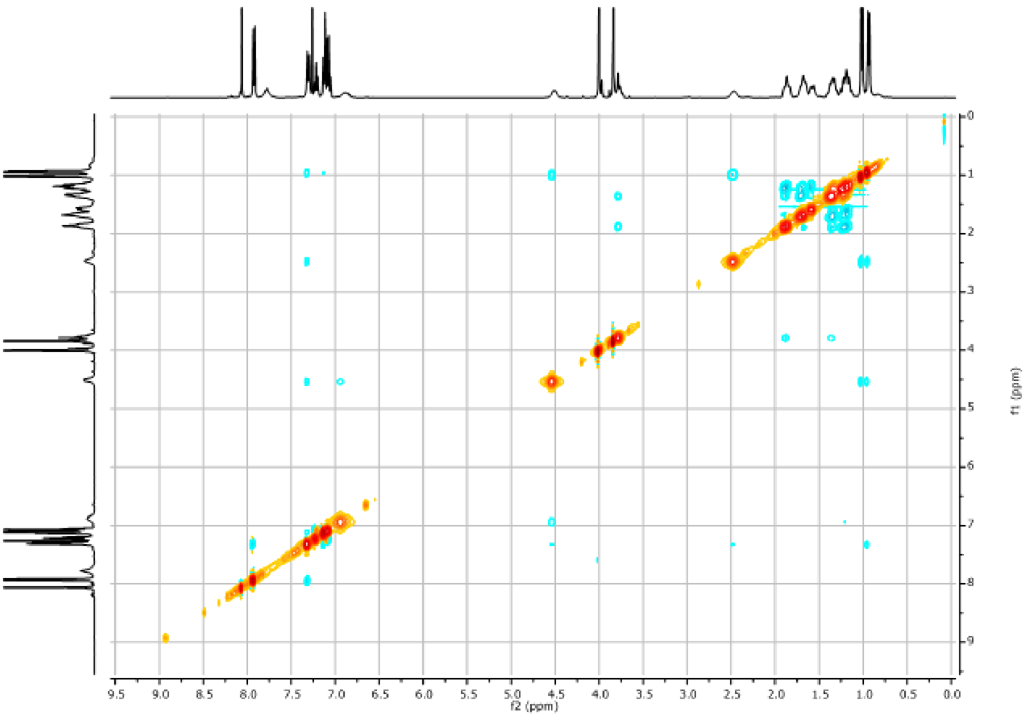

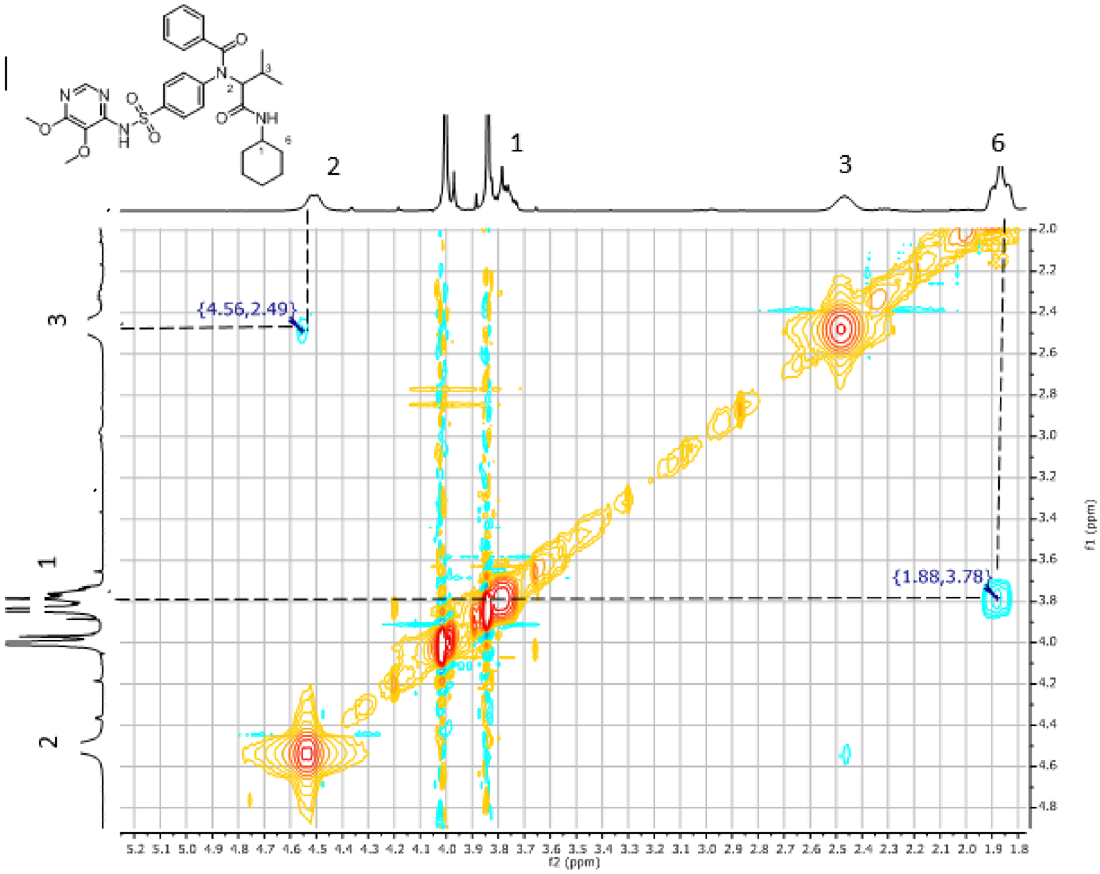

***N*-(2-(*tert*-butylamino)-1-(4-chlorophenyl)-2-oxoethyl)-*N*-(4-(*N*-(5,6-dimethoxypyrimidin-4-yl)sulfamoyl)phenyl)benzamide (7b)**

^{1}H-NMR (400 MHz, $CDCl_3$)

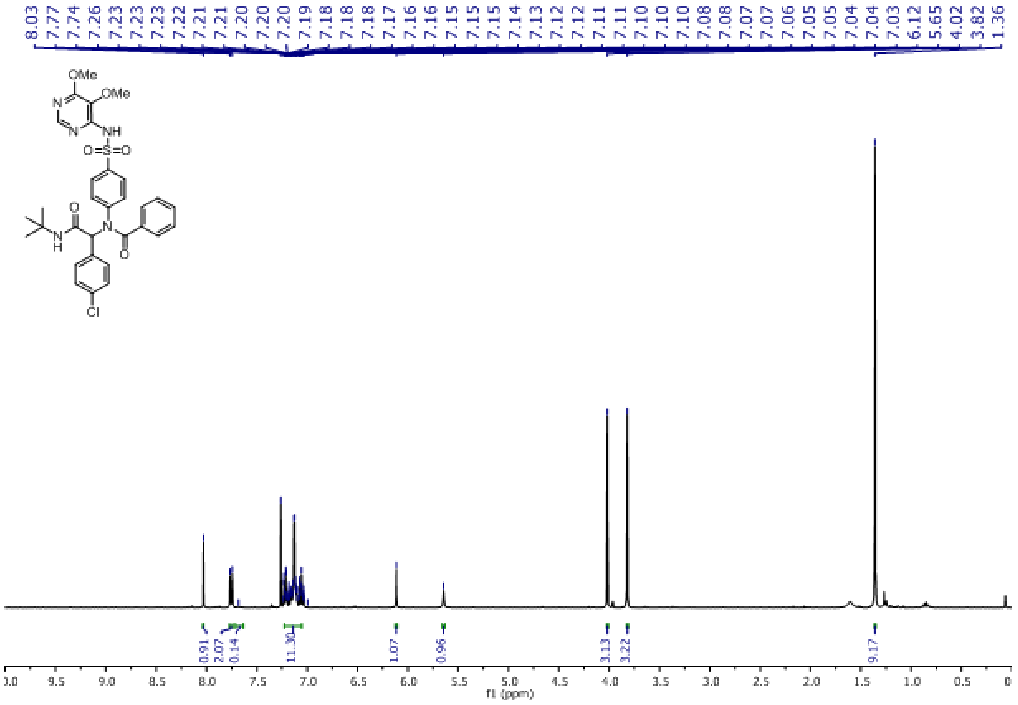

^{13}C-NMR (100 MHz, $CDCl_3$)

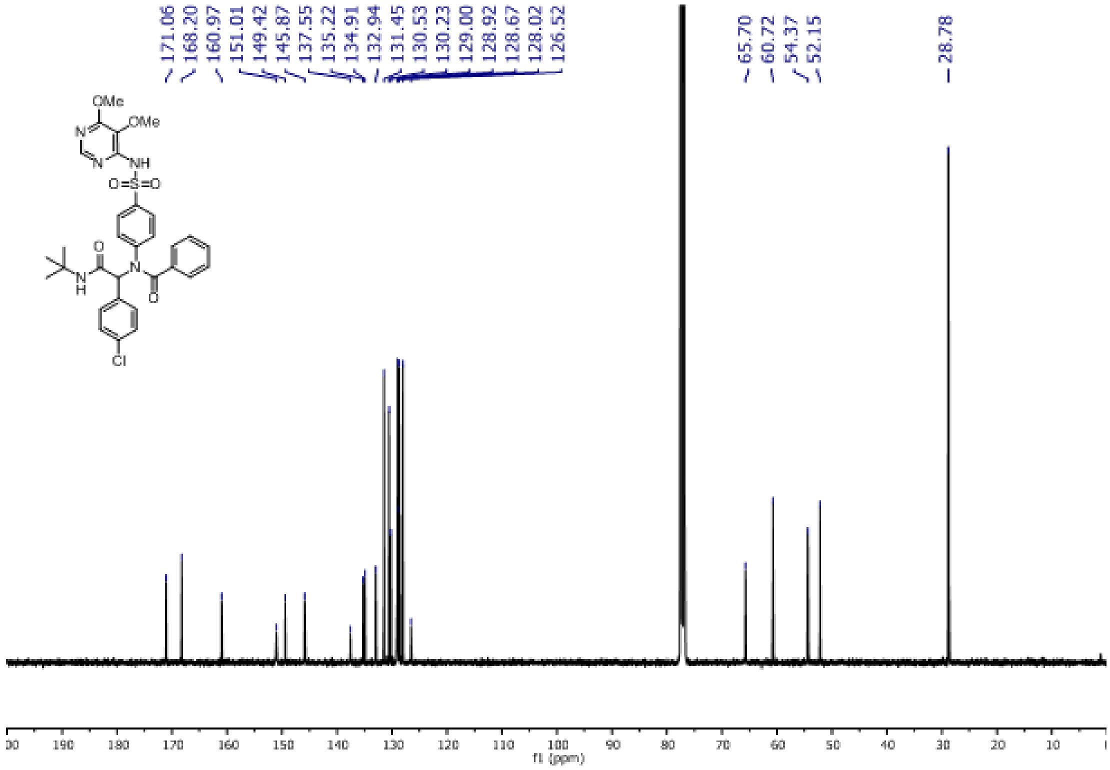

^{1}H-^{13}C HSQC

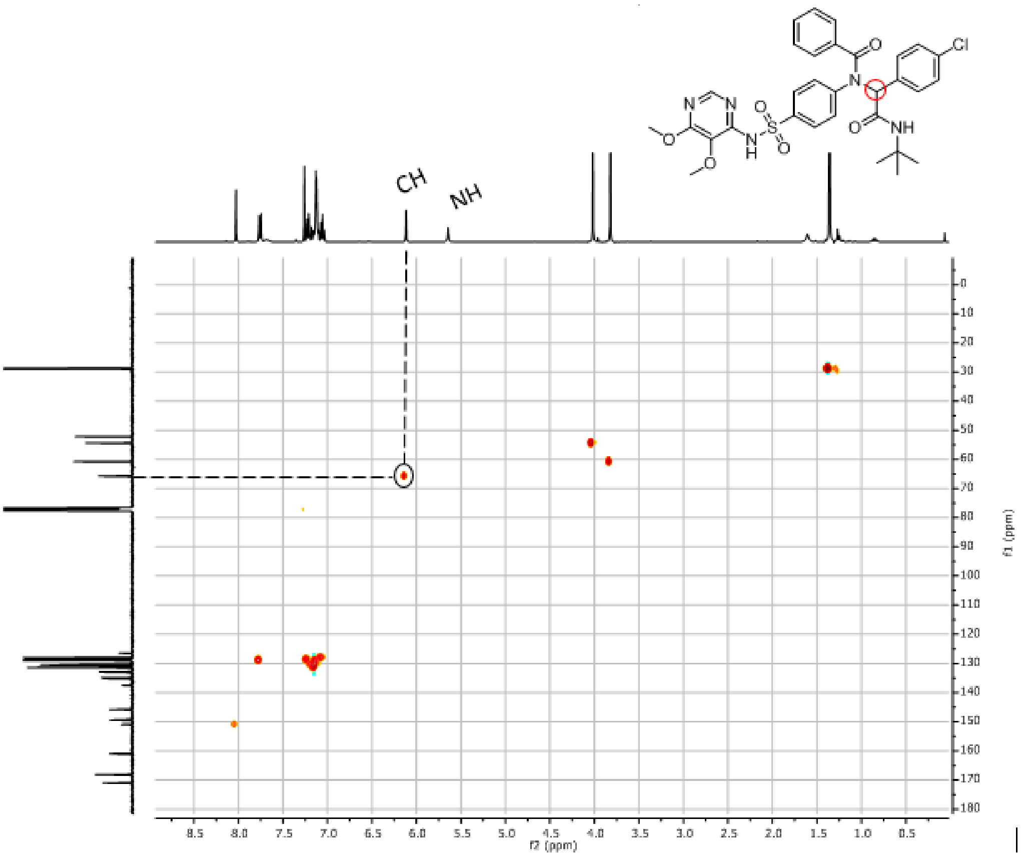

***N*-(5,6-dimethoxypyrimidin-4-yl)-2-(4-fluorophenyl)-4-phenylquinoline-6 sulfonamide (9a)**

^{1}H-NMR (400 MHz, $CDCl_3$)

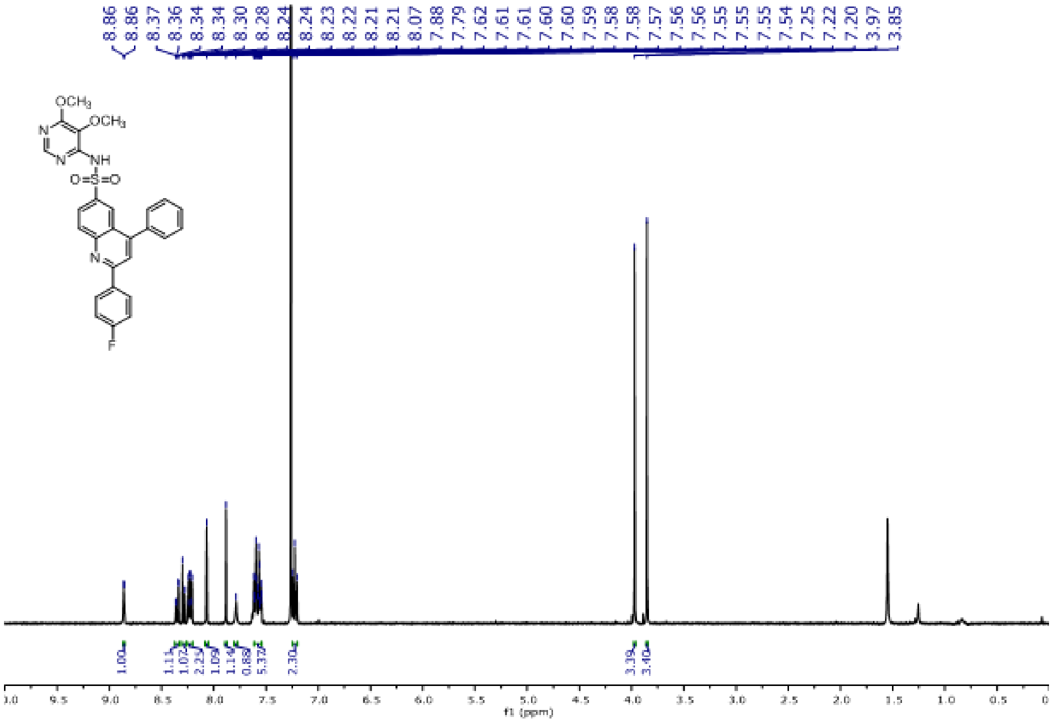

^{13}C-NMR (100 MHz, $CDCl_3$)

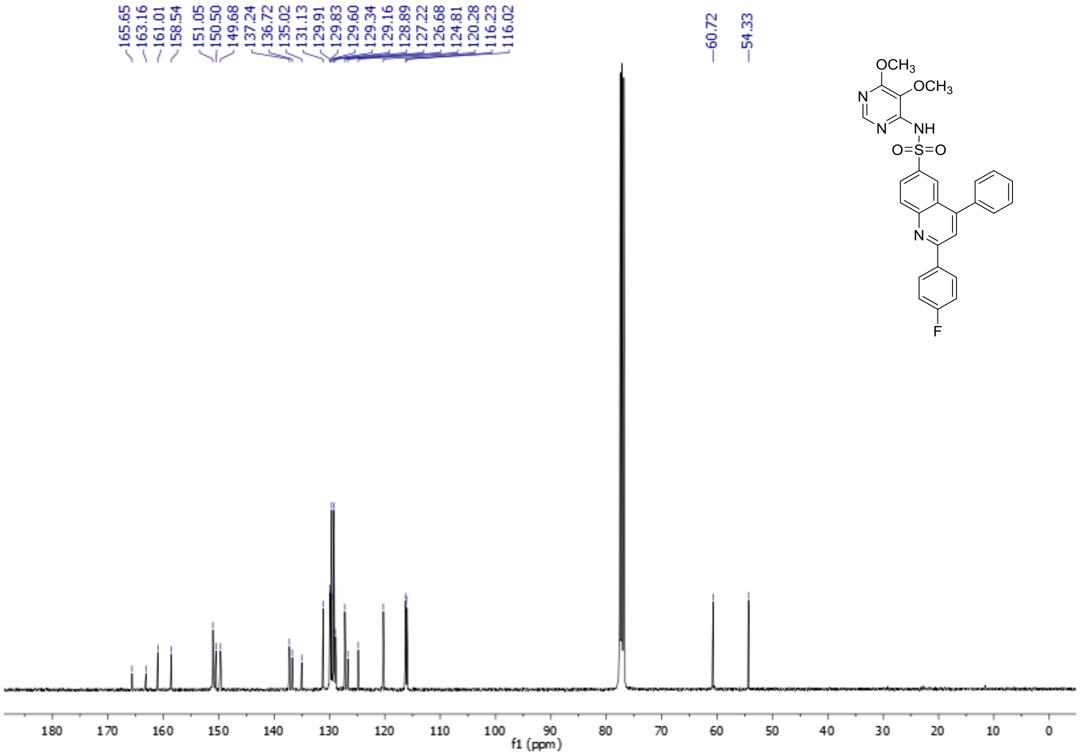

^{19}**F-NMR** (376 MHz, $CDCl_3$)

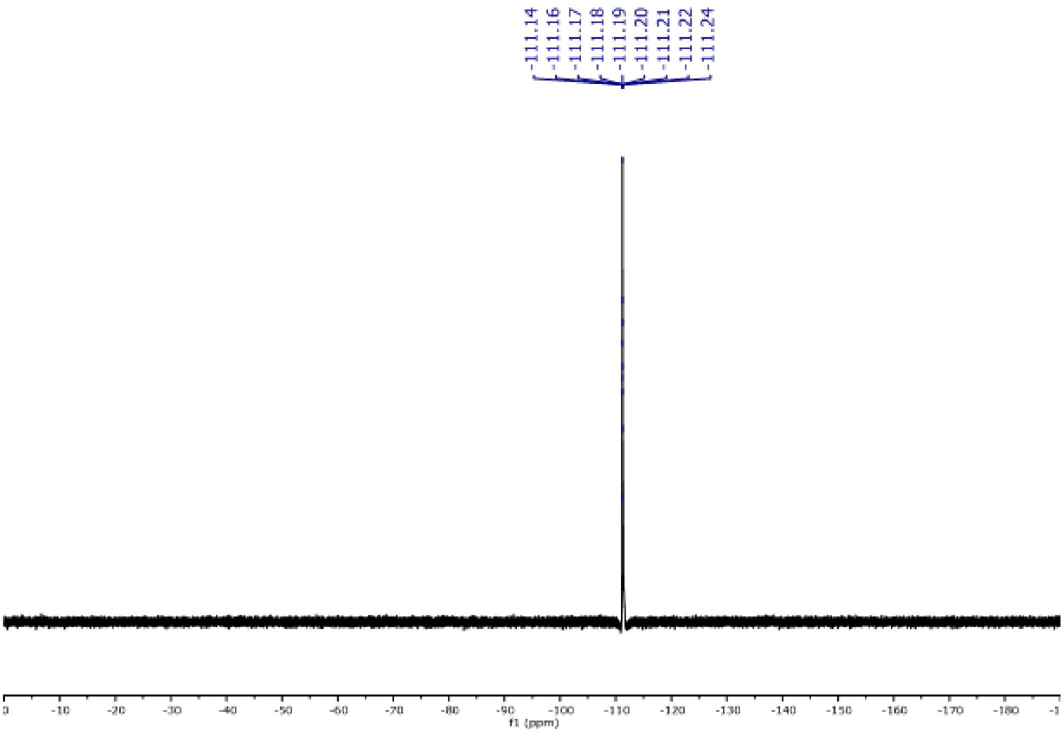

***N*-(5,6-dimethoxypyrimidin-4-yl)-4-phenyl-2-(*p*-tolyl)quinoline-6-sulfonamide (9b)**

^{1}H-NMR (400 MHz, CDCl$_3$)

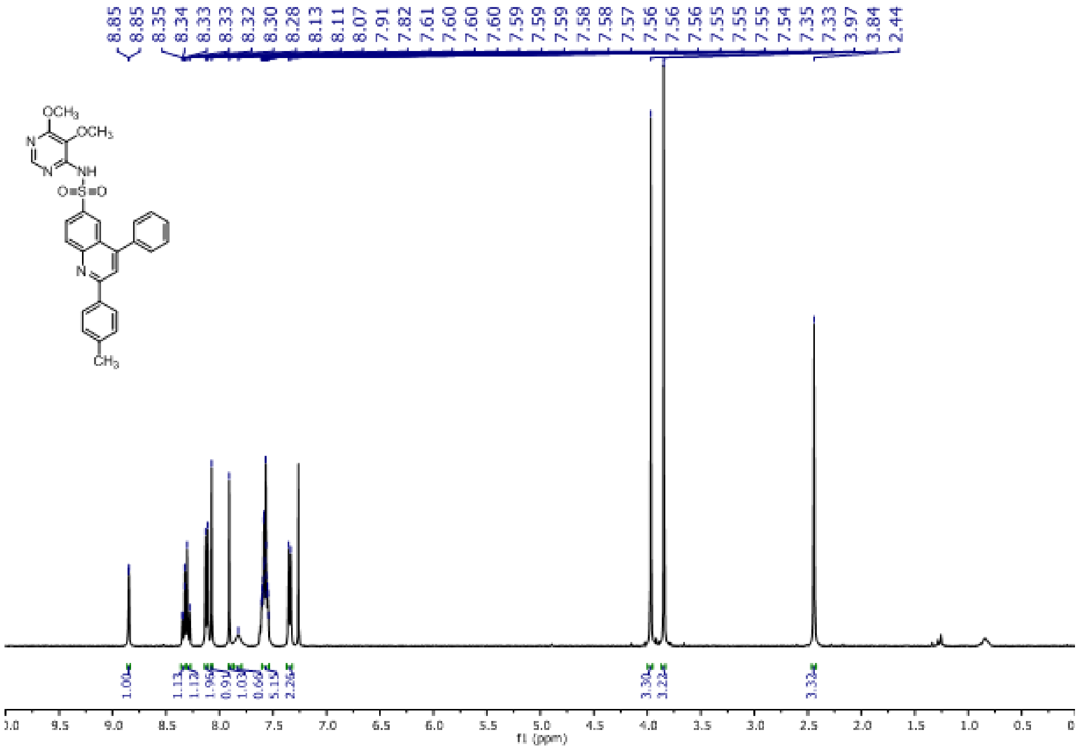

^{13}C-NMR (100 MHz, CDCl$_3$)

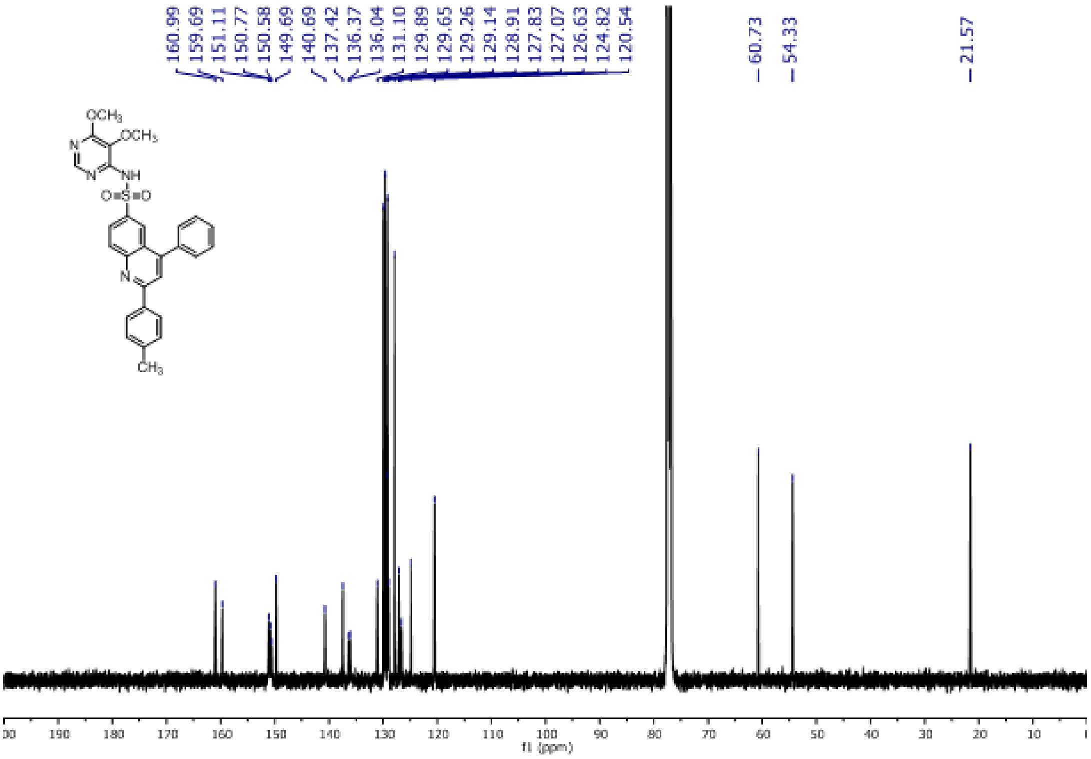

***N*-(5,6-dimethoxypyrimidin-4-yl)-4-phenyl-2-(4-(trifluoromethyl)phenyl)quinoline-6-sulfonamide (9c)**

^{1}H-NMR (400 MHz, $CDCl_3$)

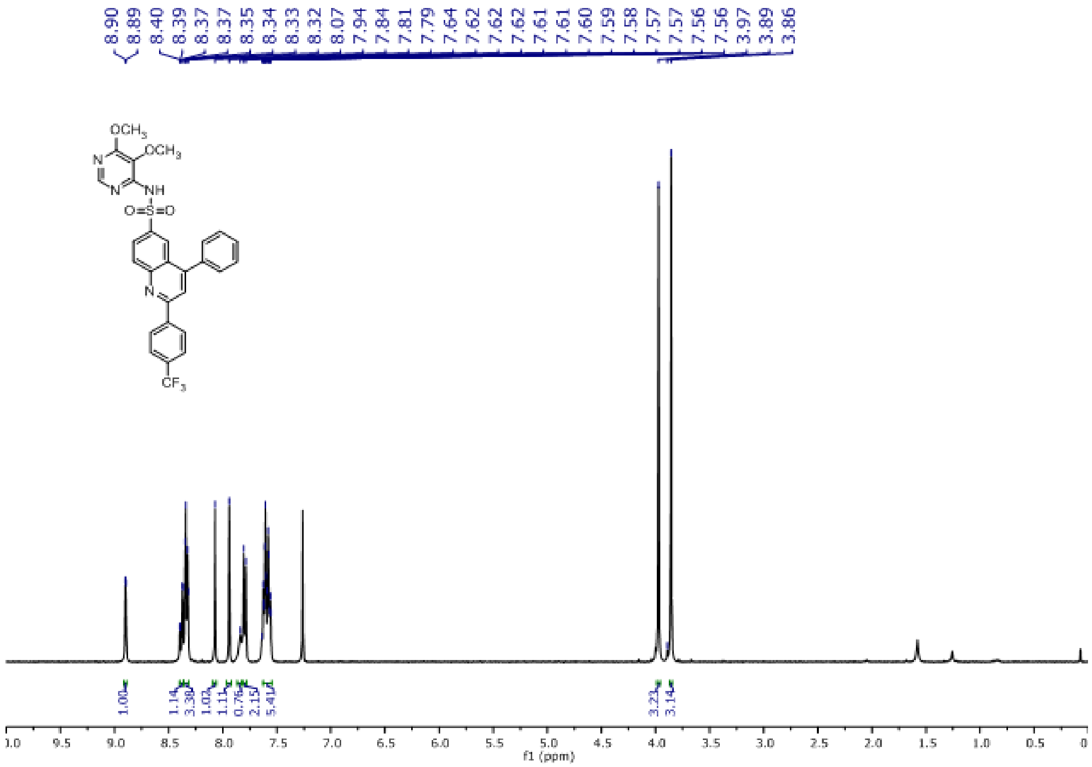

^{13}C-NMR (100 MHz, $CDCl_3$)

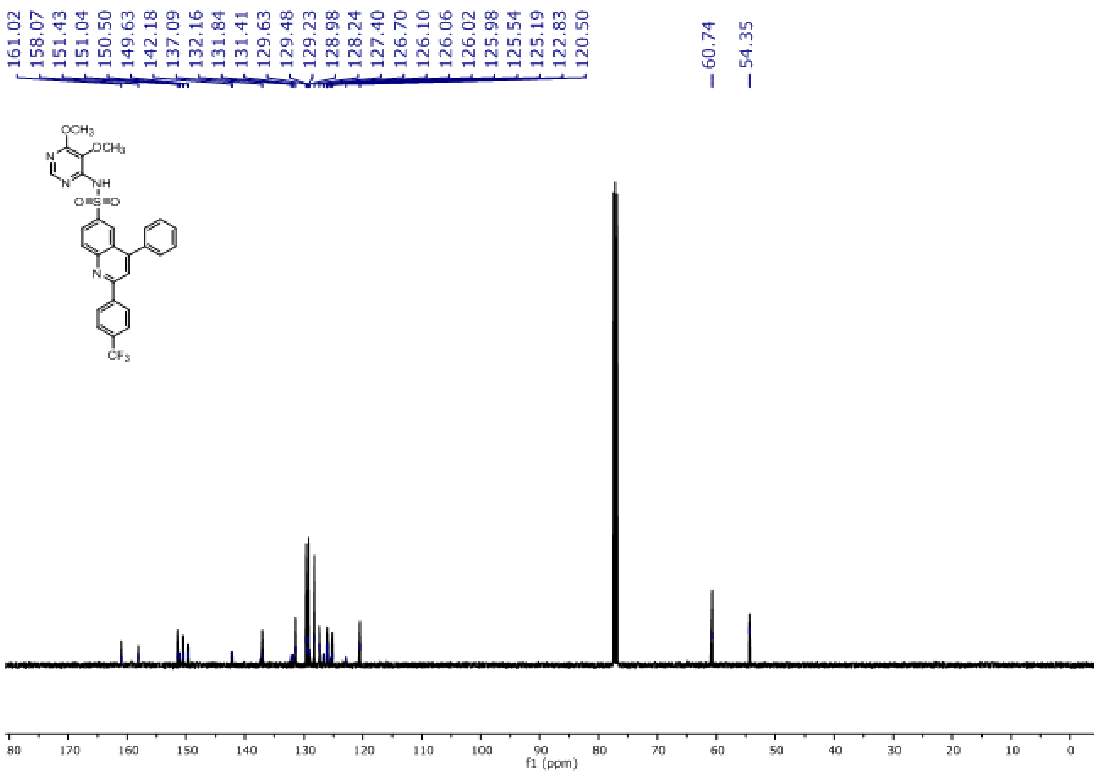

^{19}F-NMR (376 MHz, $CDCl_3$)

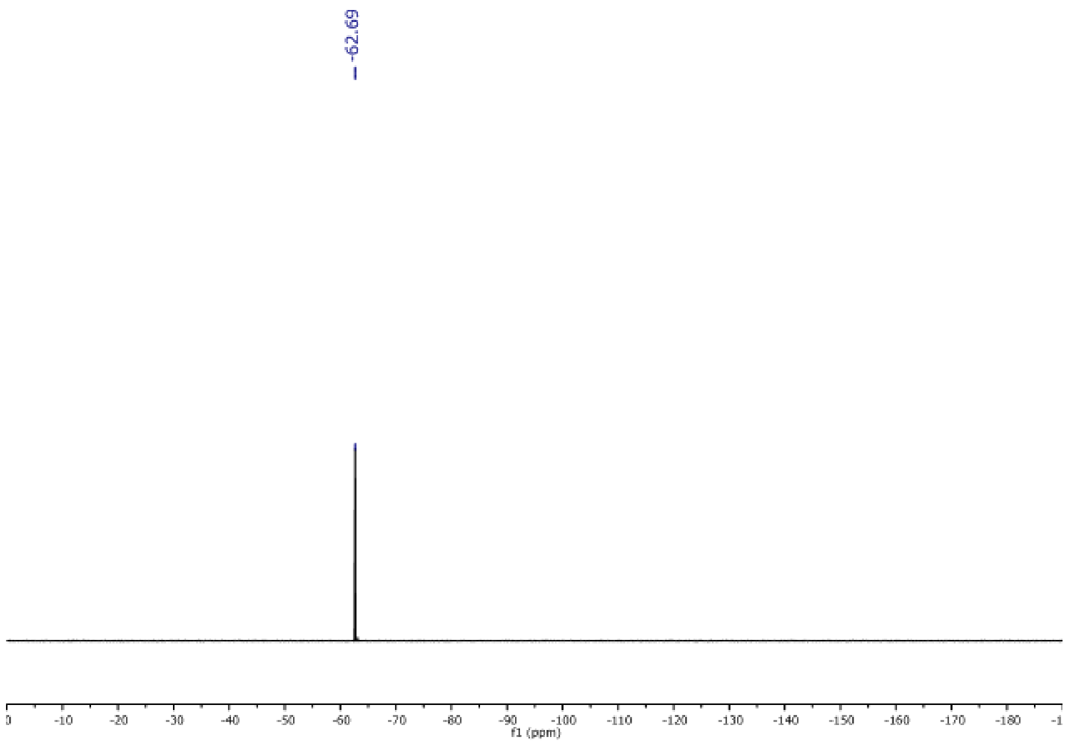

***N*-(5,6-dimethoxypyrimidin-4-yl)-2-(4-methoxyphenyl)-4-phenylquinoline-6-sulfonamide (9d)**

^{1}H-NMR (400 MHz, $CDCl_3$)

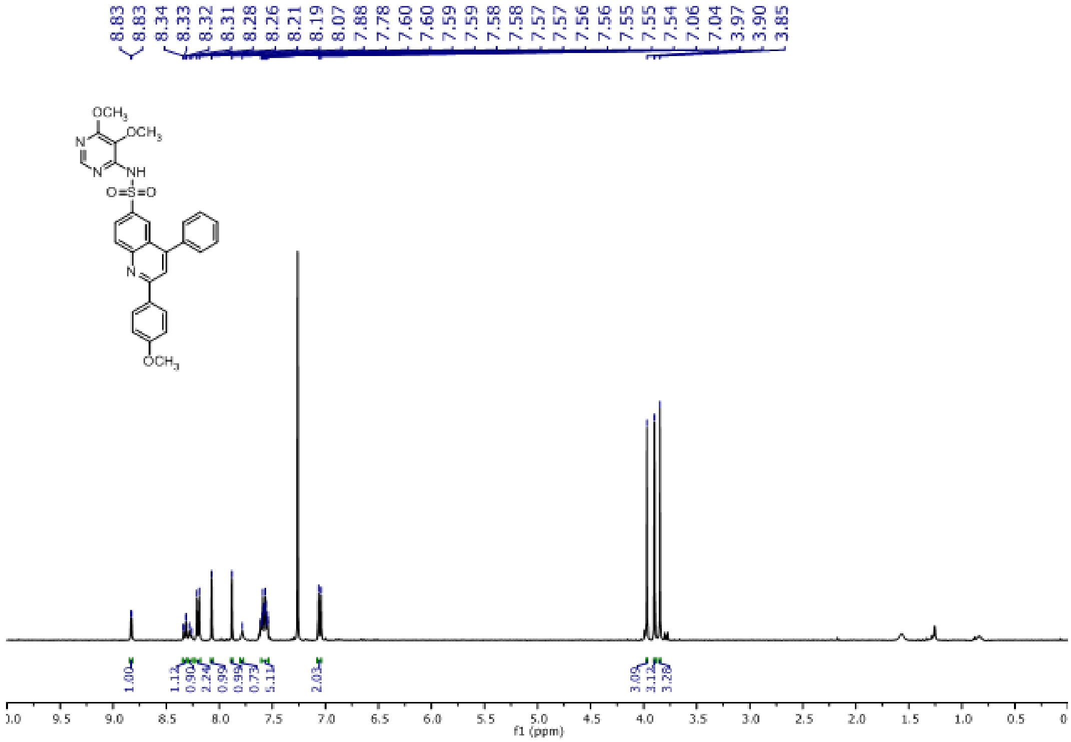

^{13}C-NMR (100 MHz, $CDCl_3$)

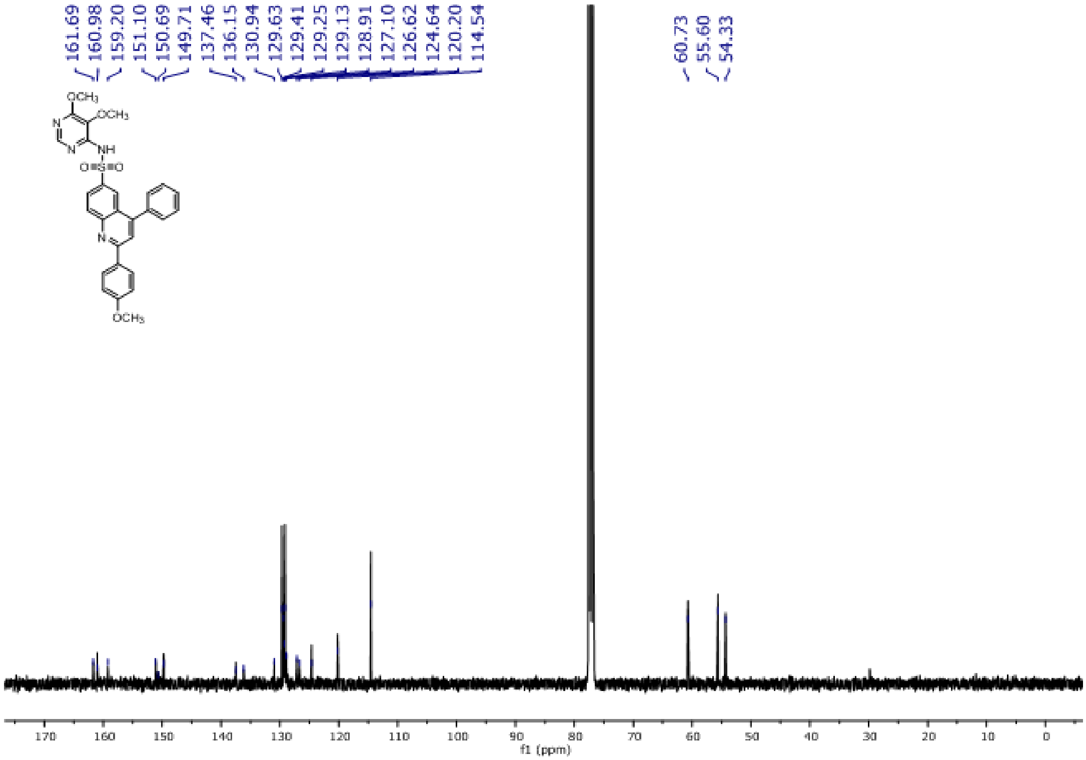

Methyl 4-(6-(*N*-(5,6-dimethoxypyrimidin-4-yl)sulfamoyl)-4-phenylquinolin-2-yl)benzoate (9e)

^{1}H-NMR (400 MHz, $CDCl_3$)

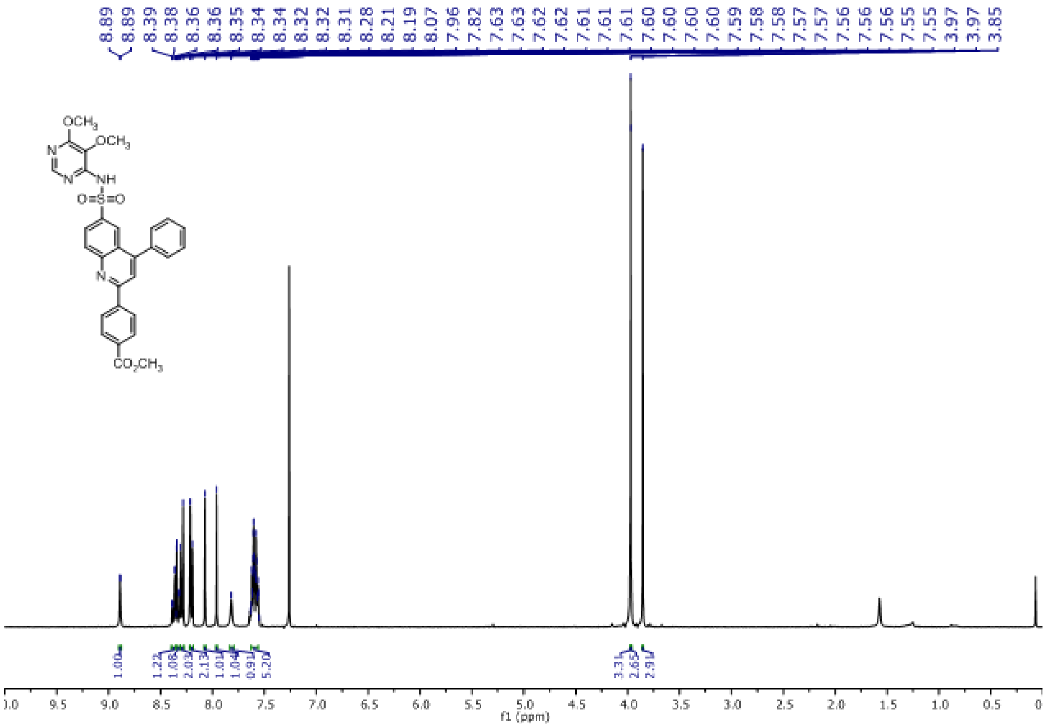

^{13}C-NMR (100 MHz, $CDCl_3$)

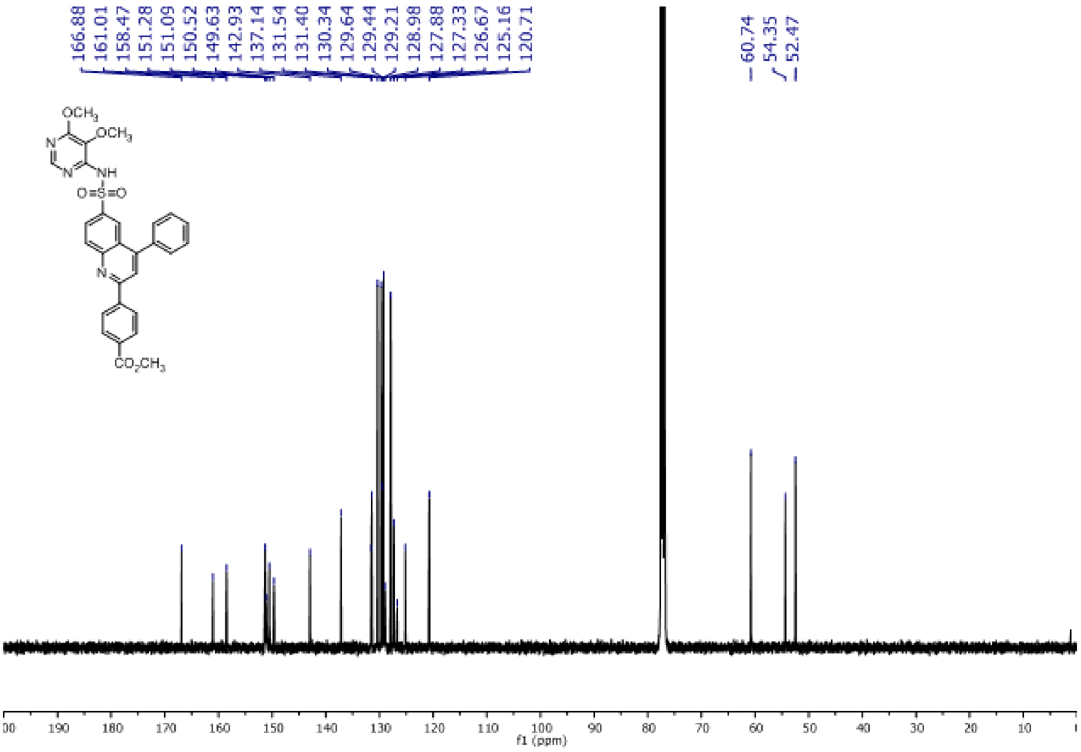

2-(4-Chlorophenyl)-*N*-(5,6-dimethoxypyrimidin-4-yl)-4-(4-fluorophenyl)quinoline-6-sulfonamide (9f)

^{1}H-NMR (400 MHz, $CDCl_3$)

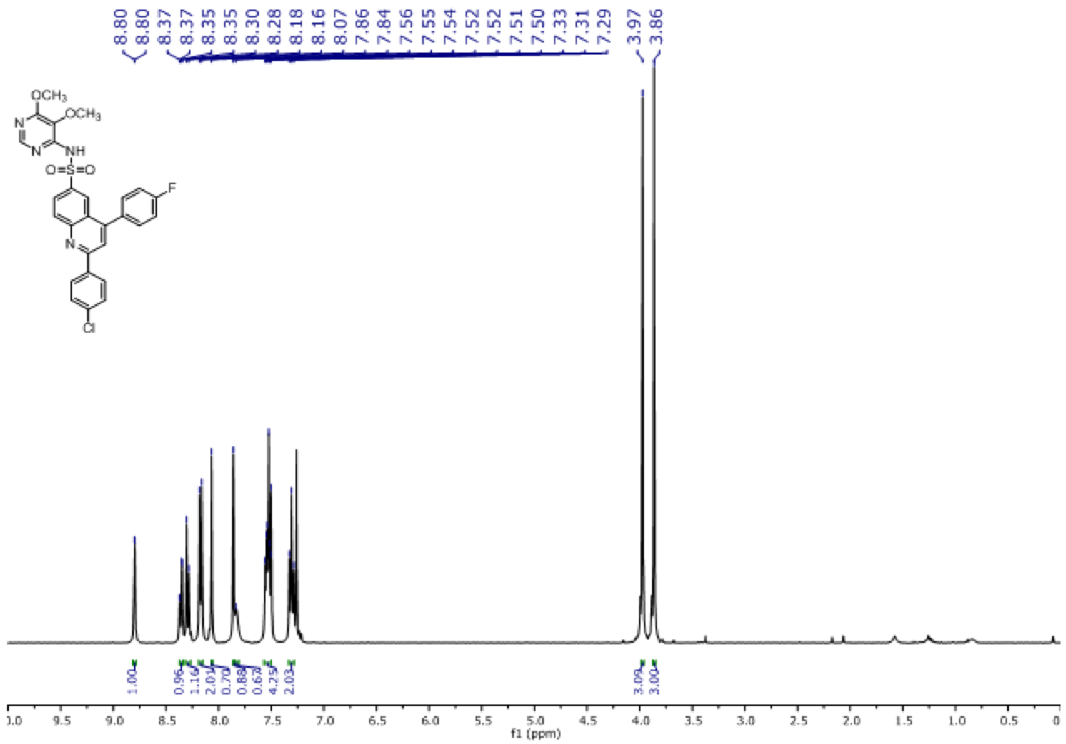

^{13}C-NMR (100 MHz, $CDCl_3$)

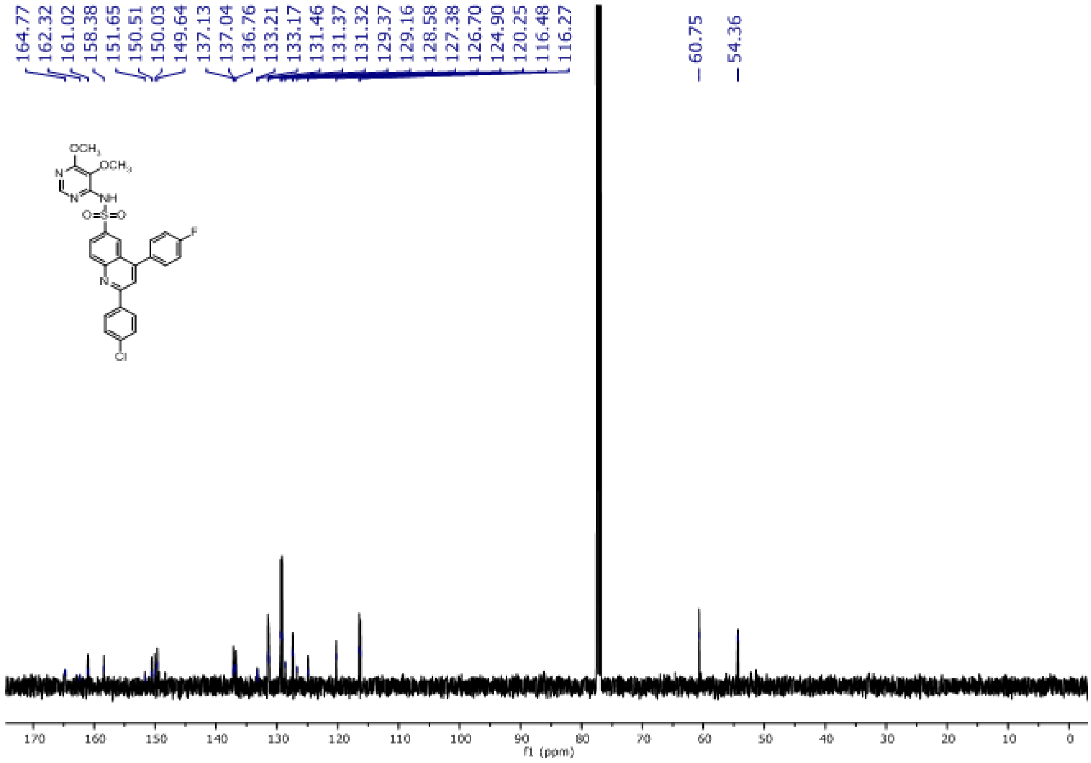

^{19}F-NMR (376 MHz, $CDCl_3$)

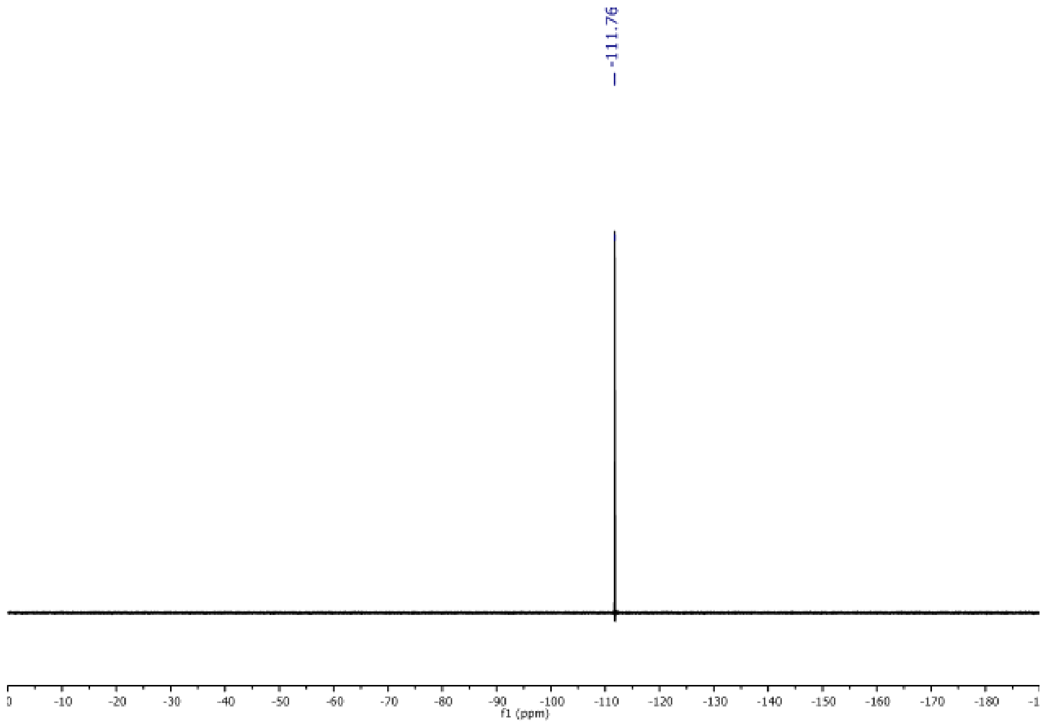

2-(4-Chlorophenyl)-*N*-(5,6-dimethoxypyrimidin-4-yl)-4-(*p*-tolyl)quinoline-6-sulfonamide (9g)

^{1}H-NMR (400 MHz, $CDCl_3$)

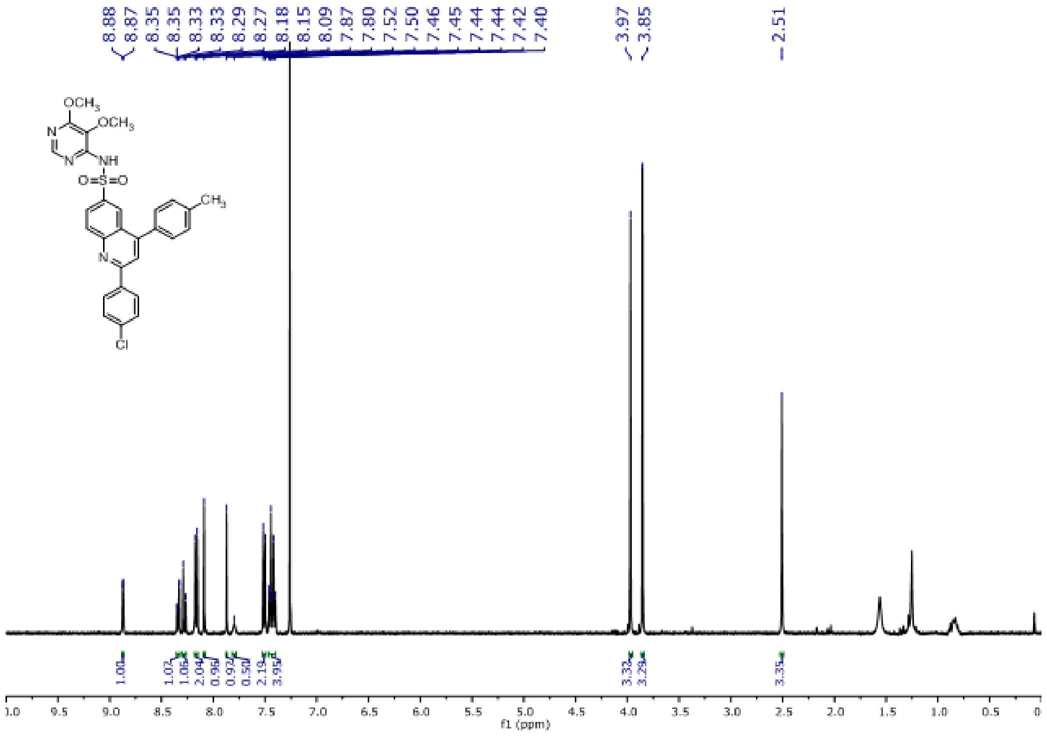

^{13}C-NMR (100 MHz, $CDCl_3$)

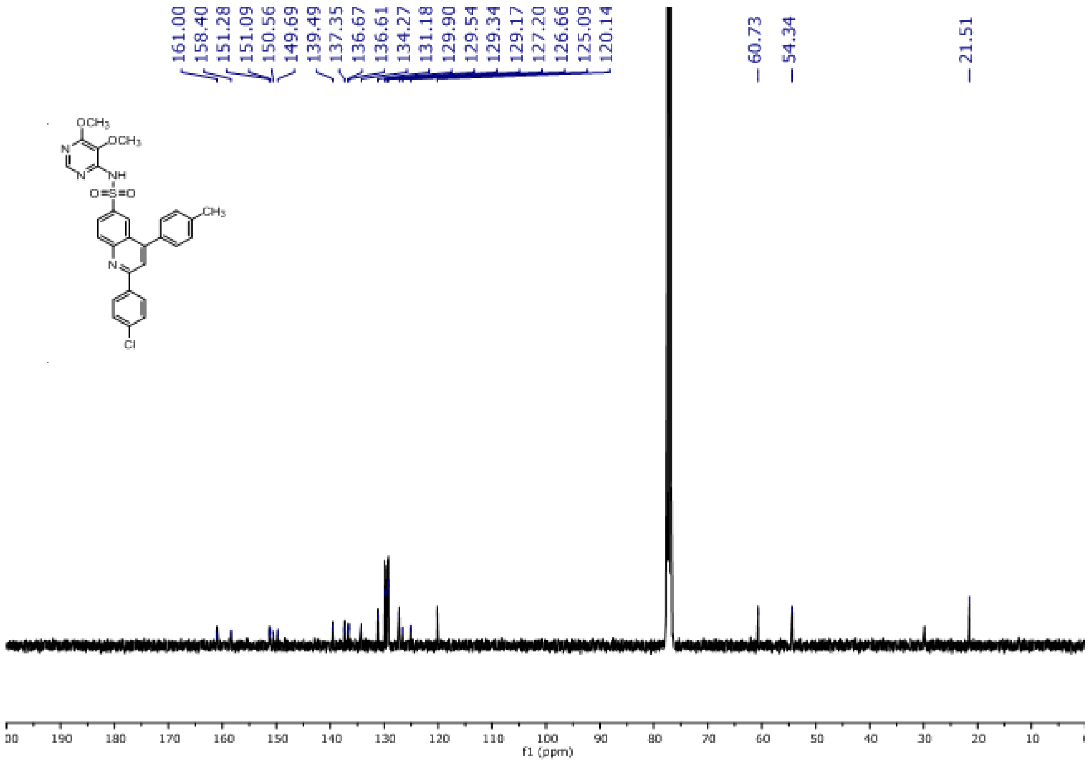

2-(4-Chlorophenyl)-N-(5,6-dimethoxypyrimidin-4-yl)-4-(4-(trifluoromethyl)phenyl)quinoline-6-sulfonamide (9h)

^{1}H-NMR (400 MHz, $CDCl_3$)

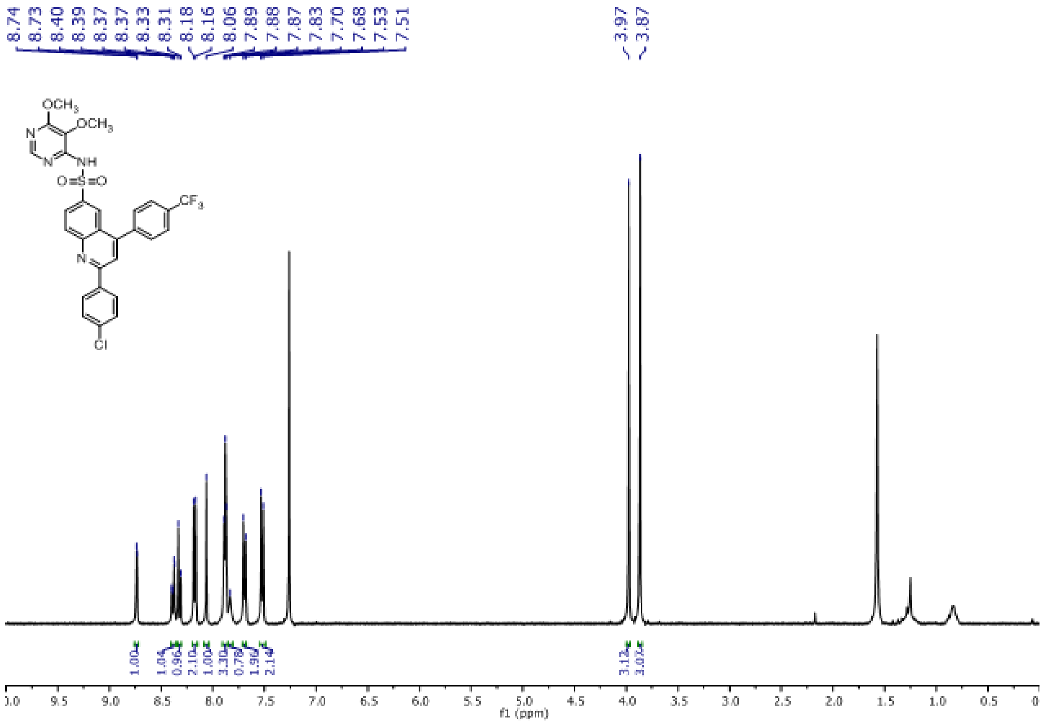

^{13}C-NMR (100 MHz, $CDCl_3$)

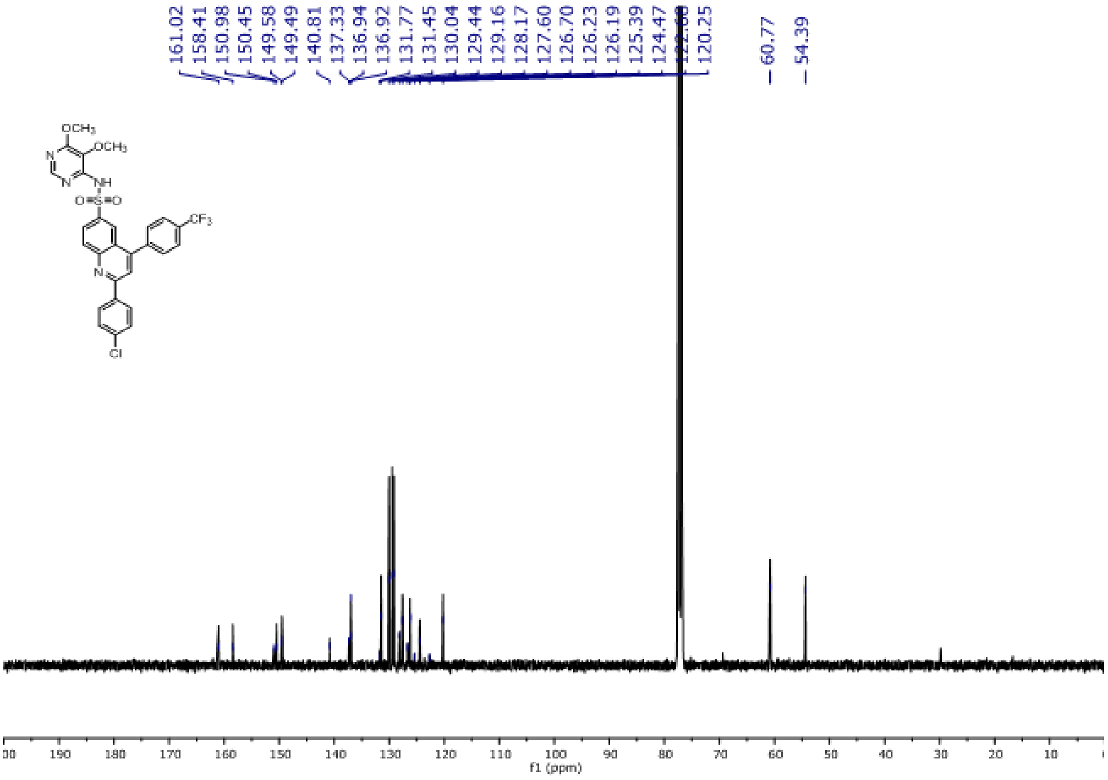

^{19}F-NMR (376 MHz, $CDCl_3$)

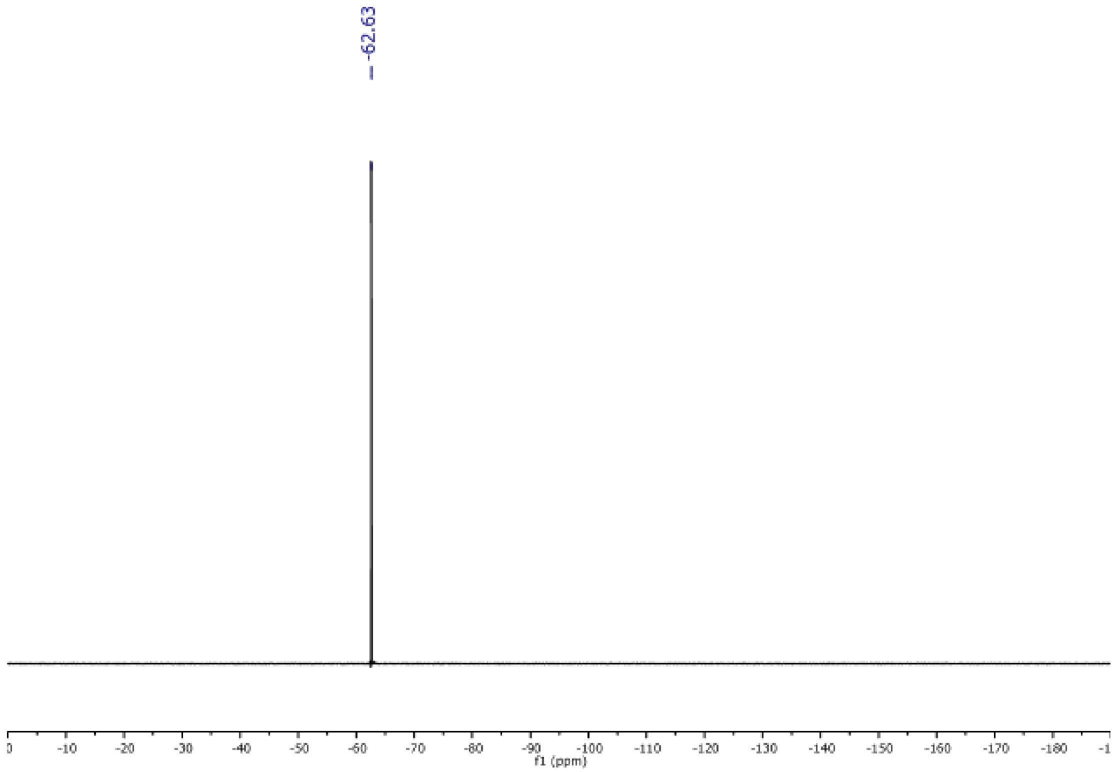

2-(4-Chlorophenyl)-*N*-(5,6-dimethoxypyrimidin-4-yl)-4-(4-methoxyphenyl)quinoline-6-sulfonamide (9i)

^{1}H-NMR (400 MHz, $CDCl_3$)

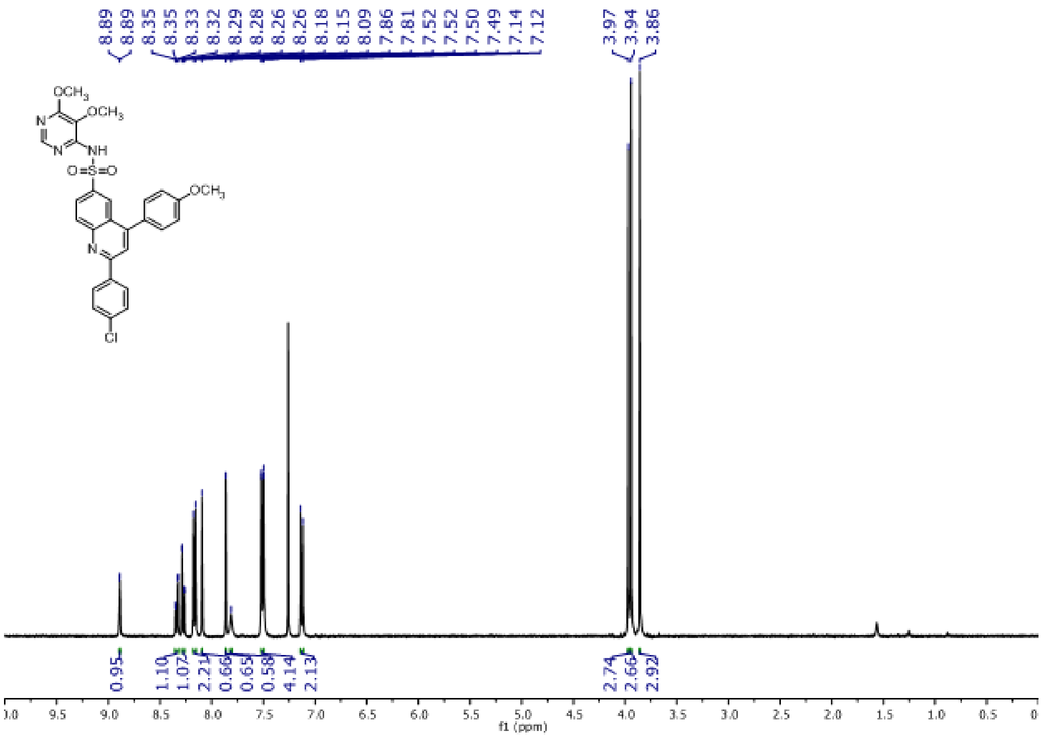

^{13}C-NMR (100 MHz, CDCl3)

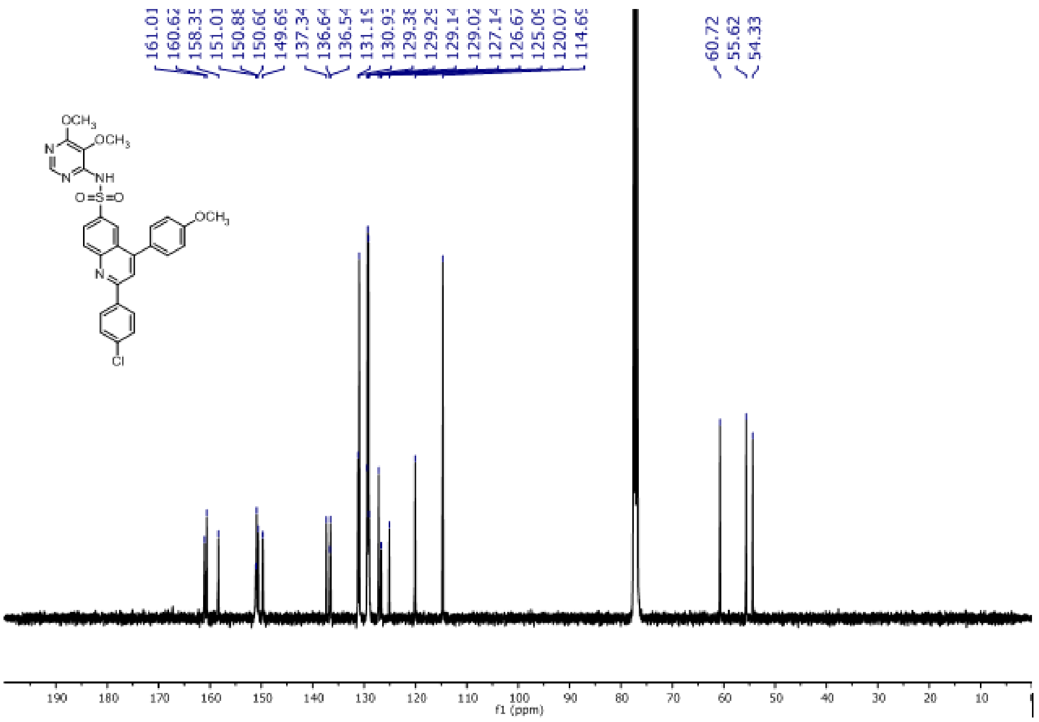

Supplement: Supplementary file 1 [file antibiotics-12-00083-s001.zip › Supporting Info I.pdf]
